# Identification of the Base-Pairing Requirements for Repression of *hctA* Translation by the Small RNA IhtA Leads to the Discovery of a New mRNA Target in *Chlamydia trachomatis*

**DOI:** 10.1371/journal.pone.0116593

**Published:** 2015-03-10

**Authors:** Nicole A. Grieshaber, Jeremiah S. Tattersall, Johella Liguori, Joseph N. Lipat, Justin Runac, Scott S. Grieshaber

**Affiliations:** 1 Department of Biological Sciences, College of Science, University of Idaho, Moscow, Idaho, United States of America; 2 Department of Oral Biology, College of Dentistry, University of Florida, Gainesville, Florida, United States of America; University of California Merced, UNITED STATES

## Abstract

The non-coding small RNA, IhtA expressed by the obligate intracellular human pathogen *Chlamydia trachomatis* modulates the translation of HctA, a key protein involved in replicative to infectious cell type differentiation. Using a combination of bioinformatics and mutagenesis we sought to identify the base pairing requirement for functional repression of HctA protein expression, with an eye to applying our findings towards the identification of additional targets. IhtA is predicted to fold into a three stem:loop structure. We found that loop 1 occludes the initiation codon of *hctA*, while loop 2 and 3 are not required for function. This 7 nucleotide region forms G/C rich interactions surrounding the AUG of *hctA*. Two additional genes in the chlamydial genome, *CTL0322* and *CTL0097*, contained some elements of the *hctA*:IhtA recognition sequence. The mRNA of both *CTL0322*and *CTL0097* interacted with IhtA in vitro as measured by biolayer interferometry. However, using a CheZ reporter expression system, IhtA only inhibited the translation of *CTL0322*. The proposed IhtA recognition site in the *CTL0322 *message contains significant G/C base pairing on either side of the initiation codon while *CTL0097* only contains G/C base pairing 3’ to the AUG initiation codon. These data suggest that as the functional interacting region is only 6-7nt in length that full translation repression is dependent on the degree of G/C base pairing. Additionally our results indicate that IhtA may regulate multiple mRNAs involved in the chlamydial infectious cycle.

## Introduction

*Chlamydiaceae* are obligate intracellular bacterial pathogens which, in a species dependent manner, infect epithelial cells of both human and animals resulting in a wide range of diseases. *Chlamydia trachomatis* is the the most common causative agent of infectious preventable blindness in developing countries [1] and the leading cause of bacterial sexually transmitted disease (STD) worldwide, infecting over 4 million people annually in the United States alone [2]. *Chlamydia* are characterized by a tightly regulated developmental cycle which begins with the infection of the host cell by the spore-like elementary body (EB). Upon infection the EB differentiates to the replicative reticulate body (RB) within a pathogen modified endocytic vacuole [3]. After multiple rounds of binary fission a subset of RBs differentiates back to the EB which in turn infect neighboring cells upon release by cell lysis or inclusion extrusion [4].

Differentiation is regulated, at least in part by HctA, a highly basic protein with primary sequence homology to the eukaryote histone H1, which binds to and densely compacts the chlamydial chromatin [5–9]. HctA is expressed late in the developmental cycle, concurrent with RB to EB conversion and has been shown to shutdown transcription and translation by modulating genome topology and/or strongly binding to DNA or RNA [6,10–14]. Expression of de novo HctA at the RB to EB transition is regulated by the small regulatory RNA (sRNA), IhtA [15]. IhtA interacts directly with the *hctA* mRNA and represses translation of the protein until the RB to EB transition point, at which time IhtA is down-regulated and HctA is expressed [15,16]. Repression of *hctA* translation by IhtA, which is itself regulated by an unknown mechanism, represents a critical "control point" in RB to EB differentiation.

Bacterial sRNAs coordinate complex biological circuits in response to many different signals by modulating protein expression. They achieve this by employing a variety of mechanisms, including but not limited to, regulating the stability or translation of their target mRNA/s (reviewed in [17]). Although sRNAs can regulate gene expression by direct base pairing or by modulating the specific activity of a regulatory protein [18], most characterized sRNAs act by direct RNA:RNA binding. Base pairing sRNAs are grouped into two broad classes; cis and trans-encoding sRNAs. The cis-encoding sRNAs display perfect complementarity to their target, while trans-encoded sRNAs, of which IhtA is an example, are encoded at a genetic location distinct from their target and generally bind their mRNA target via short interrupted base pairings [19]. Although a region of potential base pairing between a sRNA and target mRNA is typically 10–25 nucleotides, in all cases tested, only a few nucleotides are actually critical to regulation (Reviewed in [17]). It is also common for trans-encoded sRNAs to base pair with multiple mRNAs enabling a single sRNA to globally modulate a specific physiological response. For example, RyhB, a sRNA expressed by *E*.*coli*, down-regulates multiple iron-sulfur cluster containing enzymes under low iron conditions, and MicA, also expressed by *E*. *coli*, regulates multiple outer membrane porin proteins upon membrane stress. [20–22].

In this study we sought to identify the regions of both IhtA sRNA and *hctA* mRNA necessary for repression of *hctA* translation. *Chlamydia* are difficult to genetically manipulate, therefore, IhtA was originally identified using a genetic screen designed in *E*. *coli* to identify molecules involved in HctA regulation [12]. HctA expression in *E*. *coli* is lethal and IhtA was found to relieve or “rescue”ethis phenotype when co-expressed [12,15]. Here, we constructed mutants in both *ihtA* and *hctA* and assayed for loss of function when co-expressed in *E*. *coli*. We found that IhtA represses HctA expression via a 7 nt region that may provide a G/C rich clamp surrounding the start codon of the *hctA* mRNA. Further, we demonstrate that *CTL0322*, which contains this binding motif, is a novel target mRNA for IhtA.

## Results

### IhtA interacts with conserved sequences of *hctA* mRNA

**Fig 1:**
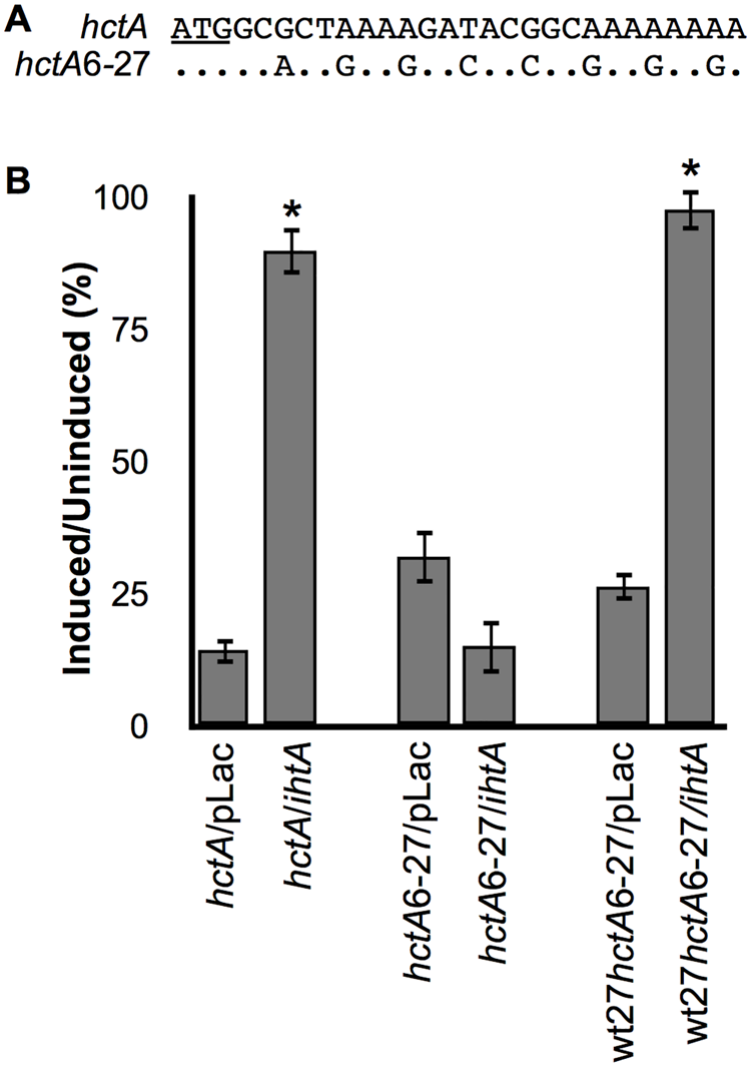
iIhtA targets conserved sequences of *hctA*. (A) Mutations made across the conserved region of *hctA* resulted in the mutant *hctA*6–27. Each mutation was designed to maintain the amino acid structure whilst disrupting the linear RNA sequence of the potential target region for IhtA. (B) Mutant *hctA* constructs were co-transformed with empty pLac vector or wild type *ihtA* into *E*. *coli* and assayed for growth upon induction of HctA expression. Wild type *hctA* co-transformed with pLac or *ihtA* served as controls. Cell viability was expressed as a percentage of the ratio between the induced and uninduced samples. Each condition was performed in triplicate over at least three separate experiments. The bars represent the mean ± SEM of the triplicates in all experiments combined. * indicates P value < 0.01 using t-test statistical analysis.

The first 31 nucleotides of the *hctA* ORF of *C*. *trachomatis* serovar L2, *C*. *trachomatis* serovar D, *C*. *muridarum*, *C*. *caviae* and *C*. *pneumoniae* are 100% conserved. Identity between *C*. *trachomatis* serovar L2 and *C*. *muridarum*, *C*. *caviae* and *C*. *pneumoniae* falls to 85%, 61% and 58% respectively outside of this region. As the sRNA IhtA is expressed and functional in the aforementioned species, and each species IhtA is capable of suppressing the translation of serovar L2 *hctA* [16], we hypothesized that IhtA interaction with *hctA* was limited to the conserved nucleotides of *hctA*. To test this hypothesis and to begin to narrow down interacting regions, we created an *hctA* mutant in which codon usage of amino acids 2–9 was changed to disrupt RNA-RNA interactions while maintaining amino acid sequence (Fig. 1A). The last codon of the conserved region codes for methionine, for which there is no alternative codon usage, therefore we focused on the first 27 nts. Expression of *hctA*6–27 in *E*.*coli* resulted in suppression of growth indicating full functionality of the mutant protein (Fig. 1B). Co-transformation of *hctA*6–27 with *ihtA* could not rescue this phenotype indicating that the sRNA IhtA could no longer functionally interact with *hctA*6–27 mRNA (Fig. 1B). In an effort to restore IhtA interaction with the mutant mRNA, we next constructed wt27*hctA*6–27 in which the first 27 nt of conserved *hctA* sequence was cloned immediately upstream of the mutant. Expression of wt27*hctA*6–27 also repressed *E*. *coli* growth indicating that this new mutant was functional. Co-transformation of *ihtA* with wt27*hctA*6–27 resulted in rescued growth compared to wt27*hctA*6–27 alone (Fig. 1B). This data indicates that sequences within the first 27 nt of the *hctA* ORF are responsible for functional IhtA targeting.

### Loop 1 is required for IhtA repression of *hctA* translation

**Fig 2:**
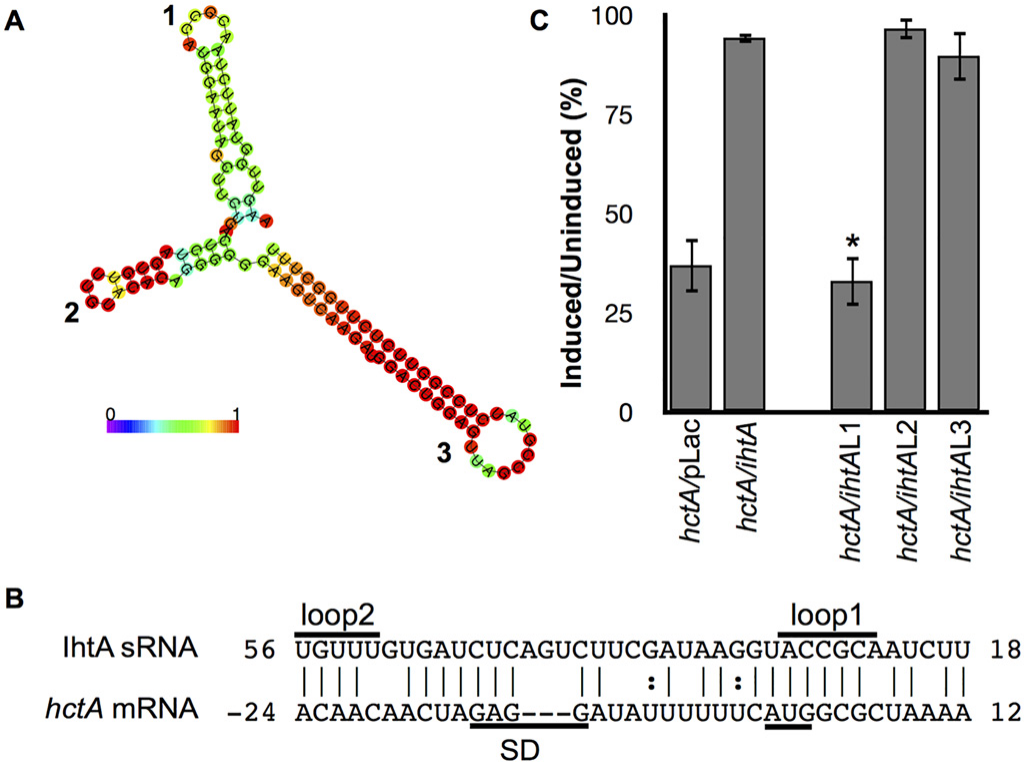
iLoop 1 of IhtA targets *hctA* mRNA. m(A) Schematic of the structure of IhtA and color coded base pair probability (color coded 1–0) as predicted by the *RNA*fold web server. The three stem:loops are indicated, stem:loop 3 is the rho-independent terminator. (B) *TargetRNA* prediction of interacting nucleotides between IhtA and *hctA*. Indicated are location of loop 1 and 2 of IhtA and the Shine-Dalgarno and start site of *hctA*. (C) Wild type *hctA* constructs were co-expressed with IhtA loop mutants *ihtA*L1, *ihtA*L2 and *ihtA*L3. Cell viability was expressed as a percentage of the ratio between the induced and uninduced samples. Each condition was performed in triplicate over at least three separate experiments. The bars represent mean ± SEM of the triplicates in all experiments combined. Statistical analysis performed using t-test, * indicates P value < 0.01 when compared to *ihtA*/*hctA*.

Structural predictions of IhtA using the RNAfold web server of the Vienna RNA Websuite [23], indicate a secondary structure of three stem:loops, the third of which is a rho-independent terminator (Fig. 2A). Typically, sRNAs interact with their targets via highly accessible, single-stranded sequence stretches, such as hairpin loops [24,25]. Indeed, the *TargetRNA* program, which identifies potential sRNA target mRNAs [26], predicts that both loop 1 and loop 2 interact with *hctA* as part of a 29 nt binding region across a 35 nt span, ranging from -24 to +12 of the *hctA* mRNA(Fig. 2B). This region also includes potential binding to the Shine Dalgarno of *hctA*. That loop 1 and 2 of IhtA are highly conserved by both sequence and structure among chlamydial species, and that the species IhtAs are interchangeable in rescue assays, increases the likelihood that these single stranded loop regions are involved in functionality [16]. To determine if one or more of these loops were required for function, we mutated loop 1, 2 and 3 of IhtA (S1 and S2 Figs.). Each mutant was co-transformed with *hctA* in an *E*. *coli* rescue assay and growth was compared to that of *hctA/*pLac and wt *ihtA/hctA E*. *coli* strains. Only the mutant sRNA IhtAL1 lost function compared to wt IhtA and could not rescue the lethal phenotype induced by expression of the HctA protein (Fig. 2C). Mutants sRNAs IhtAL2 and IhtAL3 were fully functional and are therefore not required for translation repression of *hctA* mRNA.

### IhtA occludes the AUG start site of *hctA*

**Fig 3:**
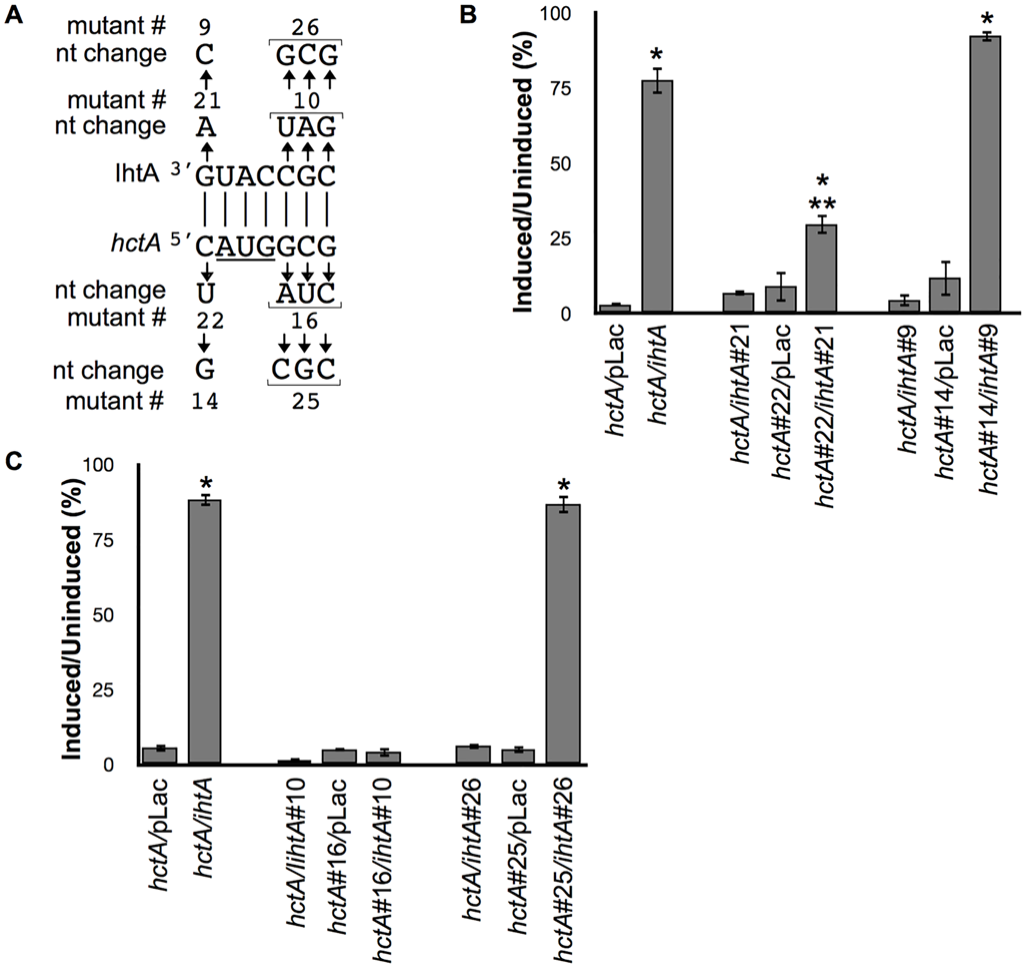
iIhtA occludes the start site of *hctA*. (A) Schematic of the mutations made in IhtA and the corresponding compensatory mutations made in *hctA*. The start site of *hctA* is underlined. (B) *E*. *coli* were co-transformed with A/T rich mutant construct pairs *ihtA*#21 and *hctA*#22 and with G/C rich mutant pairs *ihtA*#9 and *hctA*#14 and cell viability was assayed. Wild type *hctA* co-transformed with pLac, *ihtA*, *ihtA*#21 or *ihtA*#9, and mutant *hctA*s co-transformed with pLac served as controls. (C) To determine importance of the G/C pairing 3’ of the *hctA* AUG, *E*. *coli* were co-transformed with A/T rich mutant construct *ihtA*#10 and its compensatory *hctA* partner *hctA*#16 or with G/C rich mutant *ihtA*#26 and the compensatory mutant *hctA*#25. Wild type *hctA* co-transformed with pLac, *ihtA*, *ihtA*#10 or *ihtA*#26, and mutant *hctA*s co-transformed with pLac served as controls. Cell viability in graphs B and C were expressed as a percentage of the ratio between the induced and uninduced samples. Each condition was performed in triplicate over at least three separate experiments. The bars represent the mean ± SEM of all samples. Statistical analysis using t-test, * indicates P value < 0.01 when compared to the relevant *hctA* control and ** indicates P value < 0.01 when compared to *ihtA/hctA* control.

The *TargetRNA* program indicates that a 7 nt stretch largely located in loop 1 of IhtA is complementary to a 7 nt region surrounding the *hctA* start site (-1 to +6, Fig. 2B). Interaction with this region raises the possibility that IhtA may target and occlude the start site of *hctA* mRNA to prevent translation. To explore this hypothesis we constructed *ihtA* mutants focusing on sequences on either side of the anti-AUG (Fig. 3A). As the AUG of *hctA* would likely not provide target specificity, mutants were not made in the corresponding IhtA sequence. Construct *ihtA*#21, which should produce a mutant IhtA unable to interact with the “C” 5’ of the *hctA* AUG, could not rescue growth rates when co-expressed with *hctA* (Fig. 3B). To verify that this result was due to disruption of interaction, we constructed a compensatory mutant in *hctA*, *hctA*#22 which should interact with and be repressed by IhtA#21 (Fig. 3A). Induction of *hctA*#22 alone resulted in growth repression levels similar to wt *hctA* indicating that the mutant HctA#21 protein was expressed and fully functional. Co-transformation of *ihtA*#21 with *hctA*#22 resulted in slight but statistically significant growth rescue compared to *hctA*#22 alone, however rescued growth rates did not approach the control of *hctA* co-transformed with *ihtA* suggesting that *hctA*#22 may only partially compensate for the *ihtA*#21 mutation (Fig. 3B). The mutations in *ihtA*#21 and *hctA*#22 were designed to result in A/U pairing. As the complementation was weak and IhtA#21 was expressed at comparable levels to wt IhtA (S6 Fig.), we considered that maintenance of a G/C pairing may be important. To test this hypothesis a second set of mutants in this area was designed to maintain the G/C content of the original pairing (*ihtA*#9 and compensatory mutant *hctA*#14, Fig. 3A). Like *ihtA*#21, co-transformation of *ihtA*#9 with *hctA* constructs could not rescue growth repression. Co-transformation of *ihtA*#9 with its compensatory *hctA* mutant, *hctA*#14 completely rescued growth repression, indicating that the resulting mutant RNAs could now fully interact and that the single nucleotide pairing upstream of the *hctA* AUG is necessary for function (Fig. 3B).

As G/C content proved to be a factor in the previous experiment, we created four mutants to explore the predicted “GCG” base pairings directly 3’ to the AUG of *hctA*. Mutant constructs *ihtA*#10 and its compensatory mutant *hctA*#16 were designed to be A/U rich pairings, and *ihtA*#26 and its compensatory mutant *hctA*#25 were designed to be G/C rich (Fig. 3A). Both *hctA* mutants were shown to repress *E*. *coli* growth indicating robust expression and functionality (*hctA*#16/pLac and *hctA*#25/pLac respectively, Fig. 3C). Neither constructs *ihtA*#10 or *ihtA*#26 could rescue wt *hctA* induced growth repression above *hctA*/pLac levels, again suggesting the importance of this region (Fig. 3C). Co-transformation of *ihtA*#10 and compensatory *hctA*#16 mutant constructs did not result in rescued growth above the *hctA*#16/pLac control although IhtA#10 sRNA is expressed at levels comparable to that of wt IhtA (S6 Fig.). However, co-transformation of the G/C rich mutant construct *ihtA*#26 and its compensatory mutant *hctA*#25 resulted in robust growth compared to *hctA*#25/pLac control, on par to that of wt IhtA co-expressed with wt *hctA* (Fig. 3C). Taken together, the data indicate that IhtA and *hctA* interact to occlude the start site and that the surrounding G/C content may be critical to the stability of the interaction and full repression of the translation of *hctA*.

### Maintenance of stem:loop 1 structure is critical to function

**Fig 4:**
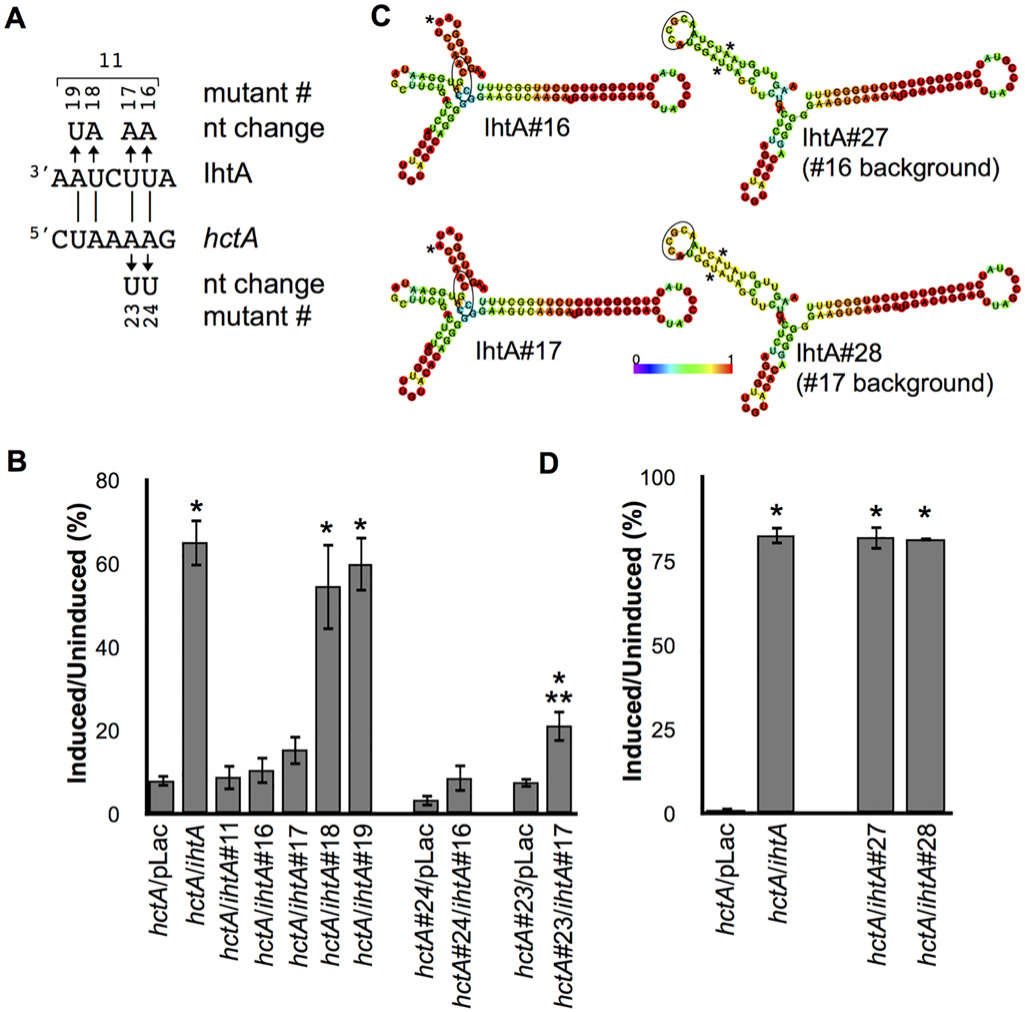
Integrity of stem:loop 1 structure. (A) Schematic of *ihtA* mutants and the corresponding compensatory *hctA* mutant. (B) Wt *hctA* was co-transformed with *ihtA* mutants #11 and #16–19 and ability to rescue growth was assayed. Wt *hctA* co-transformed with pLac and *ihtA* served as controls. Compensatory *hctA* mutants to *ihtA*#16 and *ihtA*#17 were co-transformed with the appropriate *ihtA* mutant and assayed for rescue of growth repression. *hctA*#24 and *hctA*#23 co-transformed with pLac served as baseline controls. (C) Predicted structure of mutants IhtA#16 and IhtA#17 compared to their corresponding intramolecular compensatory mutants IhtA#27 and IhtA#28 respectively. Structures and base pair probabilities (color coded 0–1) were predicted by the *RNA*fold web server. The location of the G/C rich binding site that occludes the start site of *hctA* is circled. The * marks the mutated nucleotide/s. (D) Wt *hctA* was co-transformed with the intramolecular compensatory mutants *ihtA*#27 and *ihtA*#28 and assayed for rescue of growth. Wt *hctA* co-transformed with pLac or *ihtA* served as controls. Cell growth in graphs B and D were expressed as a percentage of the ratio between the induced and uninduced samples. Each condition was performed in triplicate over at least three separate experiments. The bars represent the mean ± SEM of all samples. Statistical analysis was performed using t-test, * indicates P value < 0.01 when compared to the relevant *hctA* or mutant *hctA* control and ** indicates P value < 0.01 when compared to *ihtA/hctA* control.

The *TargetRNA* program predicted IhtA to interact with a second region within the ORF sequence of *hctA* mRNA, distinct from the AUG and surrounding G/C rich nucleotides (Fig. 2C). The four nucleotide region in question constitutes part of the predicted stem 1 structure of IhtA and is A/U rich. To determine if this region of IhtA is important for *hctA* repression, we constructed five *ihtA* mutant constructs, one that contained mutations in all four nucleotides (*ihtA#*11) and four containing a single mutation each (*ihtA#*16–19, Fig. 4A). Co-transformation of *ihtA#*11 with *hctA* resulted in growth rates similar to the *hctA* only control indicating that the mutant sRNA IhtA#11 could no longer interact with and repress *hctA* mRNA translation (Fig. 4B). Mutant constructs *ihtA#*16 and *ihtA#1*7 also could not rescue the growth defect upon co-transformation with *hctA*, indicating that each nucleotide contributes to the phenotype of the *ihtA*#11 mutant. Co-transformation of *ihtA*#18 or *ihtA*#19 with wt *hctA* rescued the growth phenotype to levels similar to that of wt *ihtA*. Compensatory *hctA* mutants to both *ihtA*#16 (*hctA*t#24) and *ihtA*#17 (*hctA*#23) were constructed to ascertain if loss of function was due to impaired binding (Fig. 4A). Both *hctA* mutants fully repressed *E*. *coli* growth indicating full functionality (*hctA*#24/pLac and *hctA*#23/pLac, Fig. 4B). Co-transformation of *ihtA*#16 and *hctA*#24 did not result in rescue of growth while co-transformation of *ihtA*#17 and *hctA*#23 resulted in slight but statistically significant growth rescue, however the rescue did not approach control levels (Fig. 4B).

As sRNA levels of IhtA#16 and IhtA#17 expressed from the two mutant constructs were similar to that of wt IhtA (S6 Fig.) and the A/U content was not perturbed, we hypothesized that these two nucleotides may function to maintain the structure of the IhtA stem 1. Indeed the predicted sRNA structures of the IhtA#16 and IhtA#17 sRNAs indicated that the integrity of stem:loop 1 may be compromised, perhaps resulting in the loss of availability of the binding sites contained in loop 1 (Fig. 4C). The predicted sRNA structures of IhtA#18 and IhtA#19, which did not lose function, appeared similar to wt IhtA (S4 Fig.). To determine if loss of function is due to disruption of structure or disruption of predicted nucleotide pairing, we constructed intramolecular compensatory mutants within *ihtA*#16 (*ihtA*#27) and *ihtA*#17 (*ihtA*#28) to restore stem 1 and loop 1 structure (Fig. 4C), Co-transformation of *ihtA*#27 with *hctA* resulted in rescue growth rates similar to that of *ihtA*, which is in marked contrast to the rescue of the parent, *ihtA*#16 (compare Fig. 4C to Fig. 4D). Likewise, co-transformation of *ihtA*#28 with *hctA* resulted in wild type rescue levels, again in contrast to *ihtA*#17 (Fig. 4D). As the original mutation to disrupt pairing with *hctA* remains present in *both* IhtA mutants, these data indicate that this region does not functionally interact with *hctA* but is instead structural in nature.

### IhtA does not interact with the Shine-Dalgarno

**Fig 5:**
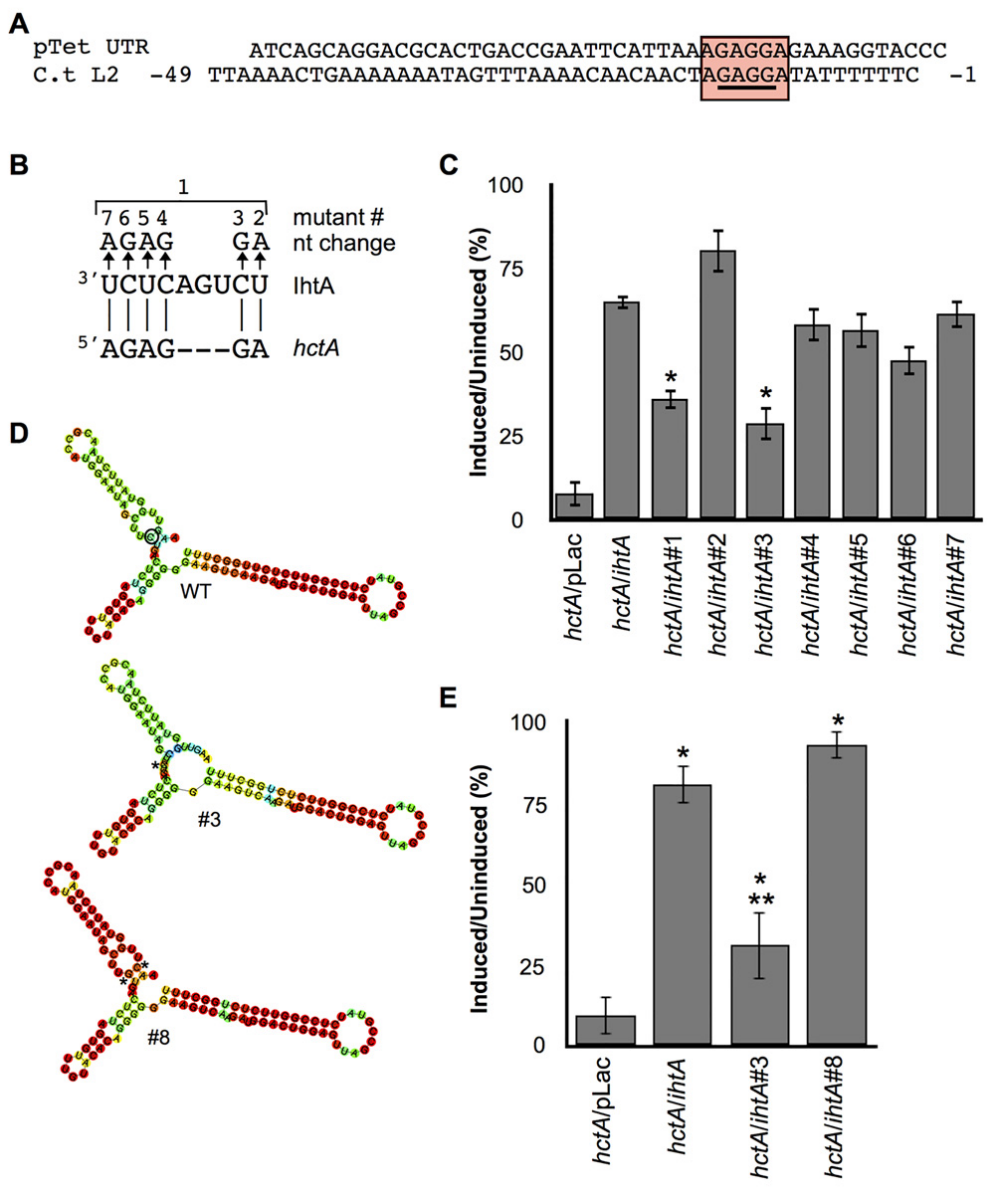
iIhtA does not interact with the Shine-Dalgarno site. (A) Schematic of the UTR of pTet compared to the 5’ UTR, starting at the TSS of *C*. *trachomatis* serovar L2. The sequences in common are boxed and the predicted serovar L2 SD site is underlined. (B) Schematic of mutations made in the anti-SD of IhtA. (C) Wild type *hctA* was co-transformed with *ihtA* mutants #1–7 and assayed for growth upon induction of HctA expression. Co-transformation with pLac and wt *ihtA* served as controls. Statistical analysis was performed using t-test, * indicates P value < 0.01 when compared to the *ihtA*/*hctA* control (D) sRNA structure and base pair probabilities (color coded 0–1) predicted by the *RNA*fold web server of IhtA#3 and its intramolecular mutant IhtA#8. The * indicates mutated nucleotide/s. (E) Cell viability of strains co-transformed with intramolecular mutant *ihtA*#8 and *hctA*. Co-transformation of *hctA* with pLac, *ihtA* or *ihtA*#3 served as controls. * indicates P value < 0.01 when compared to *hctA* control and ** indicates P value < 0.01 when compared to *ihtA/hctA*. For graphs C and E, cell viability was expressed as a percentage of the ratio between the induced and uninduced samples. Each condition was performed in triplicate over at least three separate experiments. Bars represent the mean ± SEM of all experiments combined.

We published previously that IhtA suppresses the translation of *hctA* constructs containing the ORF only (*hctA*pTet, +1 to +378) as efficiently as that of an *hctA* construct containing the native 5’ untranslated region (UTR*hctA/*pTet, -49 to +378) [15]. We concluded from that data that functional interaction and repression must occur within the ORF itself. It would, therefore, appear unlikely that the *TargetRNA* predicted interactions of IhtA to the SD region of *hctA* mRNA would occur (underlined in Fig. 2C). However, upon further analysis of the pTet vector into which all *hctA* fragments are cloned, it was noted that the “AGAGGA” of the pTet RBS is identical to RBS of *hctA* UTR (Fig. 5A). We reasoned that those sequences in common between pTet and 5’*hctA* UTR could be targets for functional IhtA interaction. To determine if interaction does occur between IhtA and *hctA* in this region, various anti-SD IhtA mutants were constructed (Fig. 5B and S3 and S4 Figs.). IhtA mutants were co-expressed with *hctA*pTet to focus only on the “AGAGGA” motif found in common between the pTet UTR and *hctA* UTR. Co-transformation of constructs *hctA* with mutant *ihtA*#1 which contained mutations across the entire anti-SD 6 nt region, did not result in total loss of function, rather the percent rescue was reduced from the control mean percentage of 65% (*hctA*/*ihtA*) to a mean percentage of 36% (Fig. 5C). As *ihtA*#1 contained multiple mutations we sought to determine if a single nucleotide or multiple nucleotides contributed to knockdown of function. Six mutants containing a single point mutation along the anti-SD sequence were constructed (Fig. 5B). Of all the mutants, only *ihtA*#3 displayed a reduction in function similar to that of *ihtA*#1 when co-transformed with *hctA* (Fig. 5C). None of the other mutant constructs resulted in a significant loss of rescue when co-transformed with *hctA* compared to the *hctA*/*ihtA* control.

The mutation in the construct *ihtA*#3 was located within the stem of stem:loop 1 (Fig. 5D). We therefore considered if structural issues could be at play in the partial loss of function as sRNA expression levels of IhtA#3 were similar to wt IhtA (S6 Fig.). As expected the multiple mutations of the sRNA IhtA#1 resulted in serious disruption of predicted structure (RNAfold web server, S4 Fig.). The point mutation in *ihtA*#3 was also predicted to result in disruption to the structure of IhtA#3 sRNA (Fig. 5D). To determine if the structural difference predicted for IhtA#3 could account for partial loss of function, an intramolecular compensatory mutant was constructed to restore structure but maintain disruption of interaction with the SD site, resulting in mutant sRNA IhtA#8 (Fig. 5D). When co-transformed with *hctA*, *ihtA*#8 rescued growth significantly compared *ihtA*#3/*hctA* and to similar levels as wt *ihtA*, indicating that disruption of structure accounted for loss of function of IhtA#3 (Fig. 5E). Taken together, these data indicate that IhtA does not repress *hctA* translation by direct interaction with the SD site and that occlusion of the AUG of *hctA* is likely sufficient to inhibit translation.

### Interaction of IhtA with additional mRNA targets containing the IhtA/*hctA* binding motif

**Fig 6:**
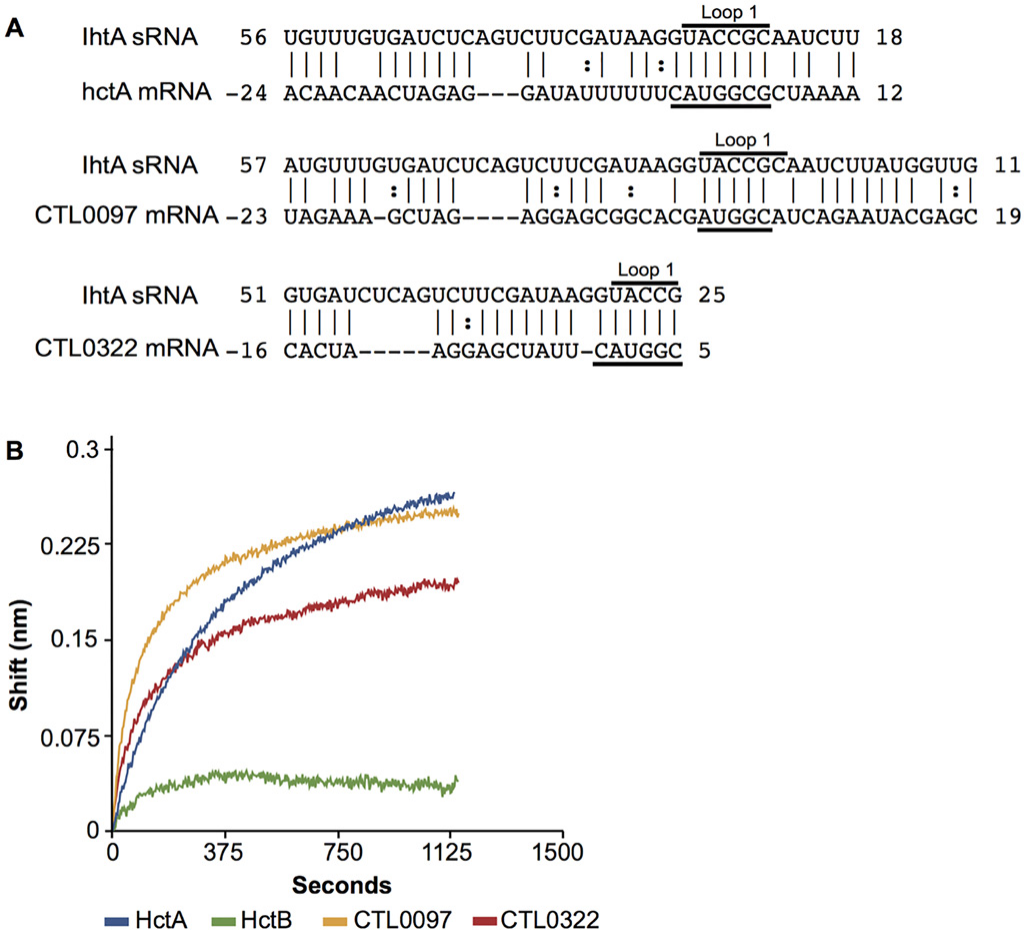
iInteraction of IhtA with predicted mRNA targets *CTL0097* and *CTL0322*. (A) Predicted base-pairing by *TargetRNA* of additional IhtA target mRNAs which encode at least a partial G/C rich IhtA binding region (underlined). The location of loop 1 of IhtA is also indicated. (B) In vitro transcribed IhtA was incubated with *CTL0322* and *CTL0097* immobilized on a biosensor tip. IhtA incubated with in vitro transcribed *hctA* served as a positive control for binding and in vitro transcribed *hctB* served as a negative control. Binding was measured as a change in internally reflected light through the tip over time.

The experiments thus far predict that IhtA occludes the start site of *hctA* and that G/C content of the interacting region is critical. We next sought to determine if IhtA regulated the expression of additional targets using a combination of 1) *TargetRNA* predictions and 2) the functional binding criteria determined for IhtA:*hctA*. *TargetRNA* predicts a total of four targets in *C*. *trachomatis* serovar L2 when the following criteria are used: a seeding hybridization of six, p value>. 01 and interaction occurs in the 5’UTR to the first 30 nt of the ORF. The predicted targets are listed here from highest score; *hctA* (Histone-like developmental protein), *CTL0097* (hypothetical protein), *rs16* (30S ribosomal protein, S16) and *CTL0322* (hypothetical protein). Of these predicted targets only *CTL0097* and *CTL0322* encodes at least a partial IhtA:*hctA* binding site and were therefor chosen for further study (Fig. 6A). To determine if IhtA interacts with *CTL0322* or *CTL0097* mRNA, we used biolayer interferometry (BLI) to measure RNA:RNA interactions in real-time [16]. As IhtA does not inhibit the translation of *hctB* mRNA, a second histone like protein expressed late in development [27], *hctB* transcript was used as a negative control for sRNA:RNA interactions. Briefly, in vitro transcribed *CTL0322*, *CTL0097*, *hctA* and *hctB* were immobilized on biosensor tips and dipped into a solution containing IhtA. As expected, IhtA did not interact with *hctB* but bound well to *hctA* transcript [16]. IhtA interacted with both immobilized *CTL0322* and *CTL0097* as measured by a change in internally reflected light (Fig. 6B), suggesting that *CTL0322* and *CTL0097* could be additional targets of IhtA.

### Translation of *CTL0322* mRNA is repressed by IhtA in vitro

The G/C base pairing surrounding the AUG of *CTL0322* and *CTL0097* are not as extensive as that of *hctA*. *CTL0322* is surrounded both 5’ and 3’ of the AUG although the sequence to the 3’ of the AUG consists of only two of the three G/C nucleotides present in *hctA* resulting in a 6 nt rather than 7 nt interaction. *CTL0097* only encodes for a G/C 3’ to the AUG and does not possess 5’ G/C rich sequence, resulting in a 5 nt interaction (Fig. 6A). We therefore sought to determine if the ability of IhtA to interact with *CTL0322* and *CTL0097* by BLI translated into repression of translation in an *E*. *coli* assay using CheZ as a reporter [28]. Chemotactic bacteria switch between smooth swimming and tumbling in place in the presence of a constant chemoattractant gradient, resulting in random migration. Strains lacking *cheZ* can no longer switch between the two movements and instead tumble in place continuously, resulting in an apparent non-motile phenotype when inoculated in low agar medium. This phenotype is rescued by endogenous expression of CheZ [29–32]. The overall design of the reporter system here is to target IhtA to a *cheZ* construct in a Δ*cheZ* strain and to assay for repression of motility rescue.

**Fig 7:**
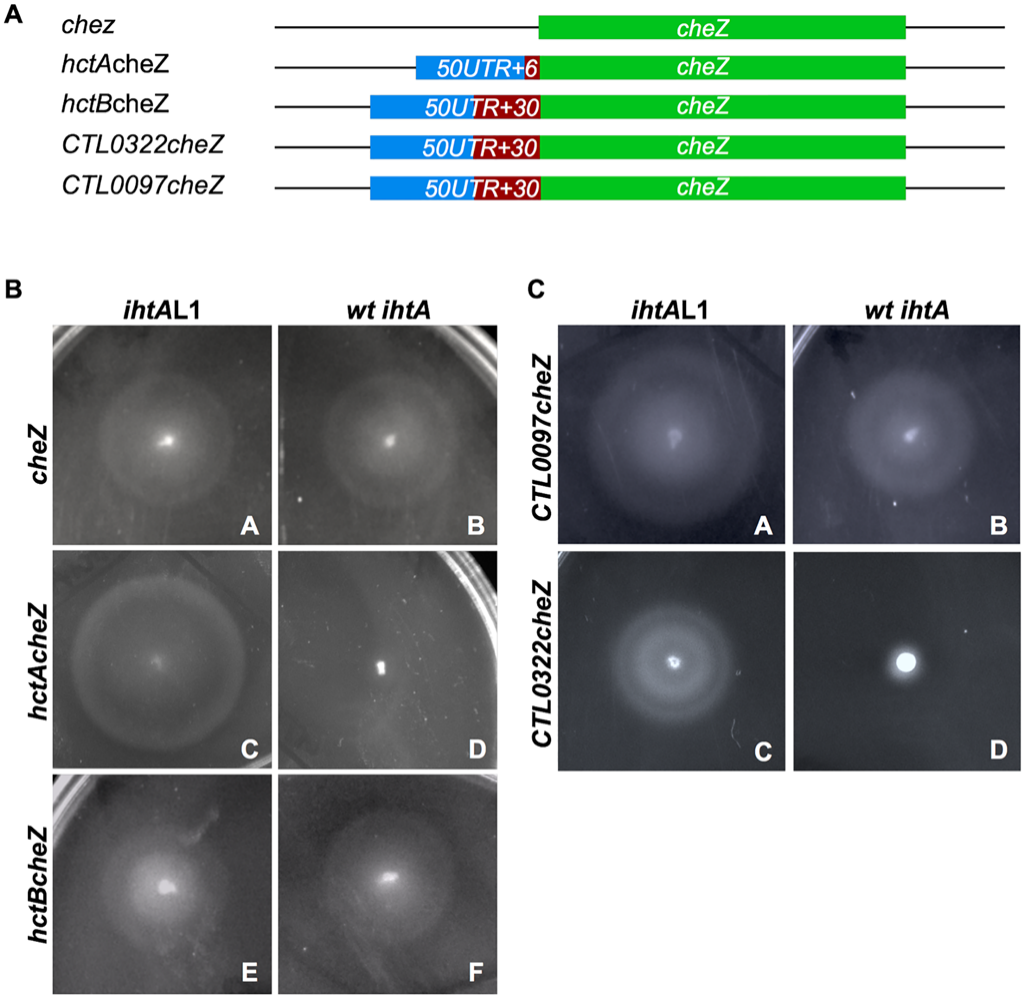
iIhtA represses the translation of *CTL0322* in vitro. (A) Schematic of *cheZ* constructs. Blue and red are chlamydial specific sequences, red indicates sequence that is translated. Green indicates *E*. *coli* cheZ. (B) *E*. *coli* lacking motility (MG1655 Δ*cheZ*) were co-transformed with either *ihtA*L1 (deficient in *hctA* translation repression) and *cheZ*, *hctBcheZ* or *hctAcheZ* (panels A,C and E) or *ihtA* and the aforementioned constructs (panels B, D and F). (C) *E*. *coli* lacking motility (MG1655 Δ*cheZ*) were co-transformed with *ihtA*L1 and *CTL0097cheZ* or *CTL0322cheZ* (panel A and C) and *ihtA* and *CTL0097cheZ* or *CTL0322cheZ* (panels B and D). All mobility assays were performed at least 3 times per strain.

To test if IhtA could target and repress expression of CTL0097 and CTL0322, PCR fragments from -50 to +30 of *CTL0322* and *CTL0097* were cloned in frame to *cheZ* as described in Materials and Methods (Fig. 7A). Each construct should transcribe an mRNA consisting of 5’ chlamydial UTR to maintain secondary structure and presentation of the targeting region, followed by 30 nt of chlamydial coding sequence in frame with *cheZ*. A fusion protein consisting of chlamydial derived sequence and CheZ will be expressed if translation is not repressed by IhtA, and, if functional, will rescue motility of the non motile MG1655 Δ*cheZ*. The construct *hctBcheZ* (-50 to +30) served as a negative control and *hctA*cheZ (-50 to +6) served as a positive control (Fig. 7A). Each construct was co-transformed with the IhtA loop 1 mutant, *ihtA*L1 (see Fig. 2), to both control for the functionality of the CheZ fusion protein and as a negative control for CheZ repression. In each case, the constructs restored motility to the MG1655 Δ*cheZ* strain indicating successful and functional fusion protein expression (Fig. 7B, panels A,C,E and Fig. 7C, panels A, C). CheZ expression from the wt *cheZ* construct was not repressed when co-transformed with wt *ihtA* as evidenced by a full motility phenotype, indicating that *cheZ* itself does not contain a cryptic IhtA targeting sequence (Fig. 7B, panel B). As a positive control for targeting of IhtA and repression of protein translation, the MG1655 Δ*cheZ* strain was co-transformed with *hctAcheZ* and *wt ihtA*. In this case motility was not rescued indicating repression of CheZ fusion protein expression (Fig. 7B, compare panel C to D). Co-transformation with *hctBcheZ* and wt *ihtA* served as an additional control as we have previously shown that IhtA does not repress *hctB* expression [27]. As anticipated, IhtA did not repress expression of the HctBCheZ fusion protein as evidenced by gain of full motility (Fig. 7B, panel D).

To determine if *CTL0322* and *CTL0097* are functional targets of IhtA, MG1655 Δ*cheZ* were transformed with *CTL0322cheZ* or *CTL0097cheZ*. Motility was rescued in both strains when the constructs were expressed alone (data not shown) or with the negative control *ihtA*L1 (Fig. 7C, panel A and C). *CTL0097cheZ* rescued motility was not repressed in strains co-expressing *CTL0097cheZ* and wt *ihtA* (Fig. 7C, panel A and B), indicating that although *CTL0097* may interact with IhtA as indicated by BLI, it is unlikely a functional target. However, *CTL0322cheZ rescued* motility was fully repressed when co-transformed with *ihtA* (Fig. 7, panel C and D). It is therefore likely that *CTL0322* is a legitimate and functional mRNA target of IhtA.

## Discussion

Understanding the mechanism by which sRNAs repress translation in *Chlamydia* is an important step in unraveling the molecular events that control the chlamydial developmental cycle. In this study we demonstrate that IhtA inhibits HctA translation by base pairing with a seven nucleotide region centered around the AUG initiation codon (C**AUG**GCG). We also show that for efficient translation repression of HctA that IhtA must form G/C base pairs on either side of the AUG codon suggesting the higher affinity bond between G/C bases is required for repression. Additionally, maintenance of the open structure of loop 1 by minimizing intramolecular interactions in the key recognition site appears to be critical to targeting *hctA*. This data suggests a model where IhtA is folded into a structure with low unfolding energy with loop 1 free to base pair with the *hctA* message. This allows IhtA to bind with high affinity to the seven nucleotide region, including the initiation codon and form key G/C bonds, likely interfering with assembly of the translation machinery.

IhtA is predicted to consist of 3 stem:loops with only 32 unpaired nucleotides out of the 107 nucleotide transcript. Of those, three regions are larger than 2 nt (loop 1, 2 and 3), with only loop 1 functional in repressing *hctA* translation. Although we did not perform structural probing experiments to validate the predicted structures of wt and mutant IhtA, our mutagenesis data combined with structure predictions suggest that the intramolecular interactions to maintain the integrity of the stem and the positioning of the targeting region is important for full function.

The mechanisms by which trans-encoded sRNAs repress mRNA translation and the requirements for binding appears quite varied in bacteria and is an active area of investigation. Many bind at or near the SD site to prevent ribosome loading, while a subset of sRNAs bind in more distal locations up or downstream, interfering with ribosome binding by other mechanisms. The most intensely studied sRNAs generally require the RNA binding protein Hfq for function and/or stability. The chlamydial genome does not code for an Hfq homolog and no protein with a parallel function has been described to date. In addition, IhtA represses HctA expression in an *hfq* deletion *E*. *coli* strain at rates similar to those when co-expressed in the parent strain (S5 Fig.). Evidence from studies of other Hfq independent sRNAs indicate that the sRNA:mRNA interaction occur over a larger region than those requiring Hfq [33–35]. IhtA, as an Hfq independent sRNA appears to buck this trend as our mutagenesis studies show that only a 7nt sRNA:mRNA duplex is required. Our data indicate however, that there is a strict requirement for the more energetically favored G/C interactions on either side of the AUG of *hctA* for translation repression to occur. These stronger interactions may compensate for the lack of a more substantial interacting region and an apparent lack of a sRNA chaperone. Additionally, the requirement for high G/C content in the background of the low G/C chlamydial chromosome may contribute to targeting specificity, as this 7 base pair binding region is present in only forty transcripts and at the 5' end of only four. Whether these genes are legitimate targets of IhtA and/or if the proximity of the binding region to the 5’ end is required for function is an area of active interest.

It is common for a trans-encoded sRNA to base pair with multiple mRNAs. Indeed, it is this characteristic that enables a single sRNA to globally modulate a specific physiological response. For example in *E*. *coli* the sRNA RyhB down-regulates multiple iron-sulfur cluster containing enzymes under low iron conditions and MicA regulates multiple outer membrane porin proteins upon membrane stress. [20–22]. To explore the possibility that IhtA regulates multiple target mRNAs to influence differentiation, a list of candidate target mRNAs predicted by *TargetRNA* was further restricted by including the functional binding region identified by our mutagenesis studies as a limiting criteria. This analysis admittedly may result in biased candidates as it is entirely possible for IhtA to interact with mRNAs over a different region than that identified to be required for repression of *hctA* translation. Nevertheless, two genes containing at least a partial IhtA recognition site, *CTL0322* and *CTL0097*, were tested as potential IhtA target mRNAs by both BLI and a functional assay. Biolayer interferometry demonstrated that IhtA could biochemically interact with both *CTL0322* and *CTL0097* at rates similar to that of *hctA*. This is perhaps not particularly surprising as IhtA is predicted to interact with 34 nt over a 42 nt region of *CTL0097* and with 21 nt over a 21 nt region of the *CTL0322* mRNA. The functional assay however, indicates that only the interactions between IhtA and *CTL0322* resulted in full repression of protein expression. It is interesting to note that neither putative target contains a perfect 7nt recognition site; *CTL0322* contains six bases of the recognition site (C**AUG**GC), while *CTL0097* contains only five (**AUG**GC). These results reinforce a model where G/C content of this minimal interacting region is important for full translation repression.

We have previously shown by Northern analysis that IhtA is expressed by 4 h post infection and is detected in purified RBs but not purified EBs. Consistent with IhtA function, HctA protein is present in purified EBs but not purified RBs. In addition, IhtA transcript decreases as RBs differentiate to EBs in a synchronized RB to EB differentiation model while HctA protein levels increase [15,36]. Microarray experiments indicate that *CT066*, the serovar D ortholog of L2 *CTL0322*, is detected by 8 h post infection and peaks by 24 h, distinct from *hctA* which is detected between 18 and 24 h [11,37]. A shotgun proteomic analysis of *C*. *trachomatis* L2, using 1-D SDS PAGE coupled with GeLC-MS/MS to identify chlamydial peptides, found CTL0322 to be present only in EBs and not in RBs isolated at 15 hours post-infection [38]. A more recent study, using LC-LC/MS-MS, found the opposite as CTL0322 was detected in RBs purified at 18 hours post infection and not in EBs [39]. It is interesting that IhtA is expressed very early in relation to *hctA*; the fact that it likely regulates at least one other target mRNA that is expressed earlier than *hctA* may provide an explanation. The dynamics and outcomes of sRNA:mRNA regulation are quite nuanced. For example most negatively acting sRNAs act in a stoichiometric manner, that is the relative ratio of sRNA to mRNA is critical. Therefore when sRNA levels are higher than the target, repression is absolute. But the sRNA has little repressive effects as target mRNA levels rise to greater than that of the sRNA. In addition, the extent and quality of interaction influences the priority of any given target, leading to titration effects when multiple targets are in play [17,40–44]. Therefore, relative abundance of transcripts, binding affinity, and presence of co-factors could all be expected to play a role in the regulation of expression by IhtA. Further research is required to fully characterize the interplay between IhtA and the *hctA* and *CTL0322* transcripts and the subsequent impact on protein expression.

Blast and Hidden Markov Model (HMM) analysis indicate CTL0322 is a hypothetical protein which contains no recognizable domains and has no homologs outside of *Chlamydia*. Similar to HctA, it is a small (18Kd), basic protein with a pI of 10.43. During the course of characterizing chlamydial specific proteins in a yeast expression system, Sisko *et al* showed that overexpression of CT066 in yeast resulted in a growth phenotype when grown under salt stress, and displayed nucleotropism when ectopically expressed in Hep2 cells [45]. Taken together, these data suggest that *CTL0322*, like HctA, may bind DNA. Although a thorough characterization of CTL0322 protein function and mRNA:IhtA kinetics remains to be investigated, that IhtA regulates the expression of at least two mRNAs, and that a single sRNA often regulates multiple mRNAs within the same physiological pathway, allows the intriguing hypothesis that IhtA regulates RB to EB differentiation by targeting multiple mRNAs involved in this critical developmental process.

Published data and estimates from a number of labs suggest there may be as many as 20–30 sRNAs expressed by *Chlamydia*. Whether a subset of these sRNAs conform to the same basic mechanism as IhtA will be an intriguing research question for the future. In particular, a better understanding of the mechanism by which chlamydial sRNAs repress protein translation may lead to sRNA-based tools for genetic manipulation, enhancing our ability to probe the molecular basis of important biological systems in these difficult to manipulate pathogens.

## Materials and Methods

### Construction and analysis of clones

All *hctA* mutants were cloned into pTet and *ihtA* mutants were cloned into pLac. The compatible vectors pTet and pLac, and constructs *hctA*pTet and *ihtA*pLac have been described elsewhere [12,15]. Mutants *hctA*6–27, wt27*hctA*6–27, *hctA*#16, *ihtA*L1, *ihtA*L2, *ihtA*L3 and *ihtA*#8 were synthesized by GenScript and provided to us in pUC57. The mutants were digested with *Kpn*I and *Pst*I and moved to pTet (*hctA* mutants) or pLac (*ihtA* mutants). All other mutants were constructed using the QuickChange II-E Site-Directed Mutagenesis Kit (Agilent Technologies) from either *hctA*pTet or *ihtA*pLac using the primers described in S1 Table. Each *hctA* mutant was tested for their ability to kill upon expression in *E*. *coli* prior to being used in a rescue assay. Comparative kill to wt *hctA* was an indication of appropriate expression and full functionality. All *ihtA* constructs were designed to encode the wt IhtA promoter sequence and should be expressed to similar levels. However a particular mutation may have affected the relative stability of the mutant sRNA. If during the course of experimentation a particular *ihtA* single point mutant construct could not rescue growth repression induced by *hctA* or the compensatory mutant *hctA*, then Northern blot analysis was performed to ascertain expression levels (S6 Fig.).

Constructs used in the CheZ mobility assay were constructed using the In-Fusion HD Cloning System (Clontech) using the primers indicated in S3 Table. The *cheZ* gene was PCR amplified using primers pTet/cheZ F and cheZ-pTet R from a *cheZ* clone kindly provided by Dr. Gullivan [28] and cloned into pTet linearized by digestion with *Kpn*I and *Pst*I. To fuse the IhtA target regions of *hctA*, *CTL0322*, *CTL0097* and *hctB* directly upstream of *cheZ*, fragments were PCR amplified from *C*. *trachomatis* serovar L2 genomic DNA and fused to linearized *cheZ*pTet which had been PCR amplified in its entirety using primers CheZ-pTet plasmidF and CheZ-pTet plasmidR. All fragments containing target sequence were designed to contain 50 nt of upstream UTR and the indicated number of ORF sequence. Each construct was tested for their ability to rescue motility in a non motile Δ*cheZ* strain to ensure expression and functionality.

### *E*. *coli* rescue assay

*E*. *coli* rescue assays were performed as previously described with minor modifications [12,16]. DH5αPRO *E*. *coli* (Clontech) cultures co-expressing the appropriate *hctA* and *ihtA* constructs were grown in triplicate overnight at 37°C in Luria–Bertani (LB) containing 100 μg/ml carbenicillin (cb), 34 μg/ml chloramphenicol (cm) and 50 μg/ml spectinomycin (spec). Cultures were then diluted 1:2000, split into two tubes and one half induced to express HctA with 100ng/ml anhydrotetracycline (aTc) and incubated with shaking at 30°C for 16 h. There is no need to induce IhtA as expression is constitutive. Growth was determined spectrophotometrically at OD_550_ and the ability of a particular construct to rescue the lethal phenotype of HctA was expressed as a percentage of the ratio between the induced (HctA expressed) and uninduced samples (normal growth).

### Statistical Analyses

Numerical data are presented as the mean ± SEM, and were analyzed by the Student’s t-test using QtiPlot software (http://qtiplot.com) or iWorks Numbers (Apple Computers).

### Biolayer interferometry

IhtA, *hctA*, *hctB*, CTL0097 and CTL0322 transcripts were synthesized from the T7 promoter of PCR amplified fragments generated from *C*. *trachomatis* serovar L2 genomic DNA using the primers described in S2 Table. All transcripts were designed to include 5’ UTR starting at the transcription start site (TSS) [46,47] and a 21nt “A” tail used to bind the transcript to the streptavidin biosensor tips. Run off transcripts were prepared using the MEGAshortscript T7 kit as described by the manufacturer (Ambion Inc.).

Biolayer interferometry studies of RNA:RNA interactions were performed using the Octet QKe (ForteBio, Menlo Park, CA) essentially as described previously [16]. To anneal the ligand (target mRNA) to the streptavidin biosensor tips (ForteBio), 150 nM target transcript, 150 nM 5’ biotinylated oligo T (complementary to the 3’ “A” tail), 1xRNA Binding Buffer (RBB, 10mM Tris-HCl pH 8, 1M NaCl, 125 mM KCl, 25mM MgCl2) were combined, heated for 1 min at 90°C and allowed to cool slowly. During this time, SA biosensor tips were equilibrated in RBB buffer for 15 min. RNA annealed to biotinylated oligo was loaded onto the streptavidin tips for 15 min or until saturation. RNA loaded tips were then soaked in RBB buffer for 5 min prior to incubation with 1500 nM IhtA. The change in internally reflected light attributable to RNA:RNA interactions was collected in real time for 20 minutes using the software provided with the Octet QKe.

### CheZ motility Assay

This assay was modified from an assay designed by the laboratory of Dr. Gullivan in which a theophylline-sensitive synthetic riboswitch regulates the translation of CheZ and thus motility in a *cheZ* knockout strain [28]. The overall design of our assay is to repress translation of *cheZ* cloned into pTet and expressed in a Δ*cheZ* strain by targeting IhtA expressed from the pLac vector to its recognition sequence fused to *cheZ*. Only if the sRNA can interact with its target sequence will translation of the *cheZ* fusion be repressed resulting in repression of *E*. *coli* motility.

We first transferred the *cheZ* knockout cassette containing kanamycin resistance from the Δ*cheZ* mutant strain, JW1870–2, generated by Baba et al. in *E*. *coli* K12 BW25113 made available as part of the Keio collection [48], to a more motile *E*. *coli* K12 strain, MG1655 (ATCC) using the method described by Datsenko et. al [49]. Briefly, a PCR fragment generated from *cheZ* mutant strain JW1870–2 genomic DNA using primers described in S3 Table was transformed into MG1655 containing the Lambda Red recombinase expression plasmid pKD46 and grown o/n at 37°C on LB agar plates containing 25 ug/ml kanamycin (kan). The resultant colonies were tested for loss of *cheZ* by both PCR and loss of motility in a soft agar assay described below. Non motile MG1655 Δ*cheZ* strains were then tested for rescue of motility upon exogenous expression of CheZ.

To test targeting of IhtA to a particular sequence, the indicated *ihtA* and *cheZ* fusion constructs were co-transformed into MG1655 Δ*cheZ*. Freshly isolated colonies were grown for 12 h at 37°C in Tryptone Broth containing 100 μg/ml cb, 34 μg/ml cm and 25 μg/ml kan. Both IhtA and CheZ are constitutively expressed in our system, therefore no induction step is required. After 12 h of growth, 2ul of each sample was inoculated into motility soft agar plates (Tryptone broth, 0.25% agar containing 100 μg/ml cb, 34 μg/ml cm and 25 μg/ml kan) and allowed to migrate o/n at RT. Transformants were considered motile if concentric rings emanating from point of inoculation in soft agar could be discerned.

## Supporting Information

S1 Fig Location and sequence of IhtA stem:loop mutations. L1, L2 and L3 indicate loop mutants.
(TIFF)

S2 Fig Predicted structures of the stem:loop IhtA mutants. Structure predictions and base pair probabilities (color coded 1–0) were calculated using the *RNA*fold web server. The predicted structure of wt IhtA is included as a reference. L1, L2 and L3 indicate loop mutants.
(TIFF)

S3 Fig Sequence of IhtA stem:loop 1 and *hctA* mutants. A) IhtA mutants in stem:loop 1. Region 1 (ant-SD), Region 2 (anti-*hctA* start site) and Region 3 (anti-*hctA* ORF) are indicated. B) Complementation mutants made in *hctA*. Regions 1, 2 and 3 are indicated. The blue colored sequence indicates pTet, the Shine-Dalgarno of which is in common with *hctA* (Region 1).
(TIFF)

S4 Fig Predicted structures of IhtA stem:loop 1 mutations. Only structures not included in the manuscript figures are represented here. Structure predictions and base pair probabilities (color coded 1–0) were calculated using the *RNA*fold web server. All mutations are indicated by an * and the location of the critical G/C clamp is circled. Wild type IhtA and the location of the open loop 1 G/C rich clamp is included as a reference structure.
(TIFF)

S5 Fig IhtA is Hfq independent. The μ*hfq* strain JW4130–1 generated by Baba et al. in *E*. *coli* K12 BW25113 and made available as part of the Keio collection [48], and the parent strain BW25113 were used to verify that IhtA is Hfq independent. Unlike the DH5αPRO *E*. *coli* strain generally used for our rescue assays, neither JW4130–1 nor BW25113 express TetR (tetracycline repressor). Therefore transformation of *hctA* should be lethal in both JW4130–1 and BW25113 as HctA will be constitutively expressed directly upon transformation and few if any colonies should grow. Co-expression of IhtA should rescue this phenotype in both strains if IhtA is Hfq independent but only in BW25113 if Hfq is required. Chemically competent JW4130–1 and BW25113 were co-transformed with either *hctA*pTet+pLac or *hctA*pTet+*ihtA*pLac. Upon transformation and growth at 37°C for an hour, the entire sample was plated on LB agar plates containing the appropriate antibiotics: JW4130–1 transformants were plated on 100 μg/ml cb, 34 μg/ml cm and 25 μg/ml kan, and BW25113 transformants were plated on 100 μg/ml cb and 34 μg/ml cm. The resulting colonies from three separate experiments were counted and graphed. JW4130–1 and BW25113 transformed with *hctA*pTet+pLac resulted in an average of 1 and 5 colonies respectively. When *hctA*pTet+*ihtA*pLac were co-transformed into the Δ*hfq* strain JW4130–1, the average number of colonies increased to 1156, approximately a 1000 fold increase. The average colony count of BW25113 co-transformed with *hctA*pTet+*ihtA*pLac also increased, from 5 to 2376, an approximately 475 fold increase.
(TIFF)

S6 Fig IhtA mutants unable to rescue wt or compensatory *hctA E*. *coli* strains are expressed at levels similar to that of wild type IhtA. IhtA mutants L1, #3, #10, #16, #17 and #21 were analyzed by Northern blot to ascertain expression levels. These mutants did not rescue wt or mutant HctA induced growth defects. The aforementioned *ihtA* mutant constructs were grown o/n in LB containing 100 μg/ml cb. *E*. *coli* expressing IhtA were pelleted and washed twice in ice cold PBS prior to sRNA isolation using the *mir*Vana miRNA Isolation kit as described by the manufacturer (Ambion, Inc.). Northern analysis was performed on sRNAs separated on a 10% TBE-urea acrylamide gel and transferred to BrightStar-Plus Nylon membrane (Ambion, Inc.). Membranes were hybridized overnight at 42°C in ULTRAhyb with a biotinylated antisense oligo probe designed against the common 3’ end of IhtA (5’ AAAGCCAAGAGAACCGGAGATACGGCTAACTCCAGTCCATCTTGACTTCCCCCCTGTGTAC 3’). The oligo was synthesized by IDT and biotinylated using a BrightStar Psoralen-Biotin Nonisotopic Labeling Kit (Ambion, Inc.). Probed membranes were washed and the IhtA species were detected with the BrightStar BioDetect Nonisotopic detection kit (Ambion, Inc.).
(TIFF)

S1 Table Oligos used to generate IhtA and hctA mutants. (DOCX)

S2 Table Primers used to generate IhtA, *hctA*, *hctB*, *CTL0097* and *CTL0322* T7 fragments for invitro transcription. tThe T7 promoter sequence is underlined. The biotinylated oligos used to immobilize hctA, hctB, CTL0097 and CTL0322 RNA to the BLI biosensor tips are also indicated.
(DOCX)

S3 Table Primers used to generate the *cheZ* deletion in strain MG1655 (CheZ Transfer, F and CheZ Transfer, R) and the clones used in the CheZ motility assay. (DOCX)
