# Supplementary figures and images for: Identification of the Base-Pairing Requirements for Repression of hctA Translation by the Small RNA IhtA Leads to the Discovery of a New mRNA Target in Chlamydia trachomatis
Source: PLoS One. 2015 Mar 10;10(3):e0116593. doi: 10.1371/journal.pone.0116593 (PMC4355289; doi:10.1371/journal.pone.0116593)

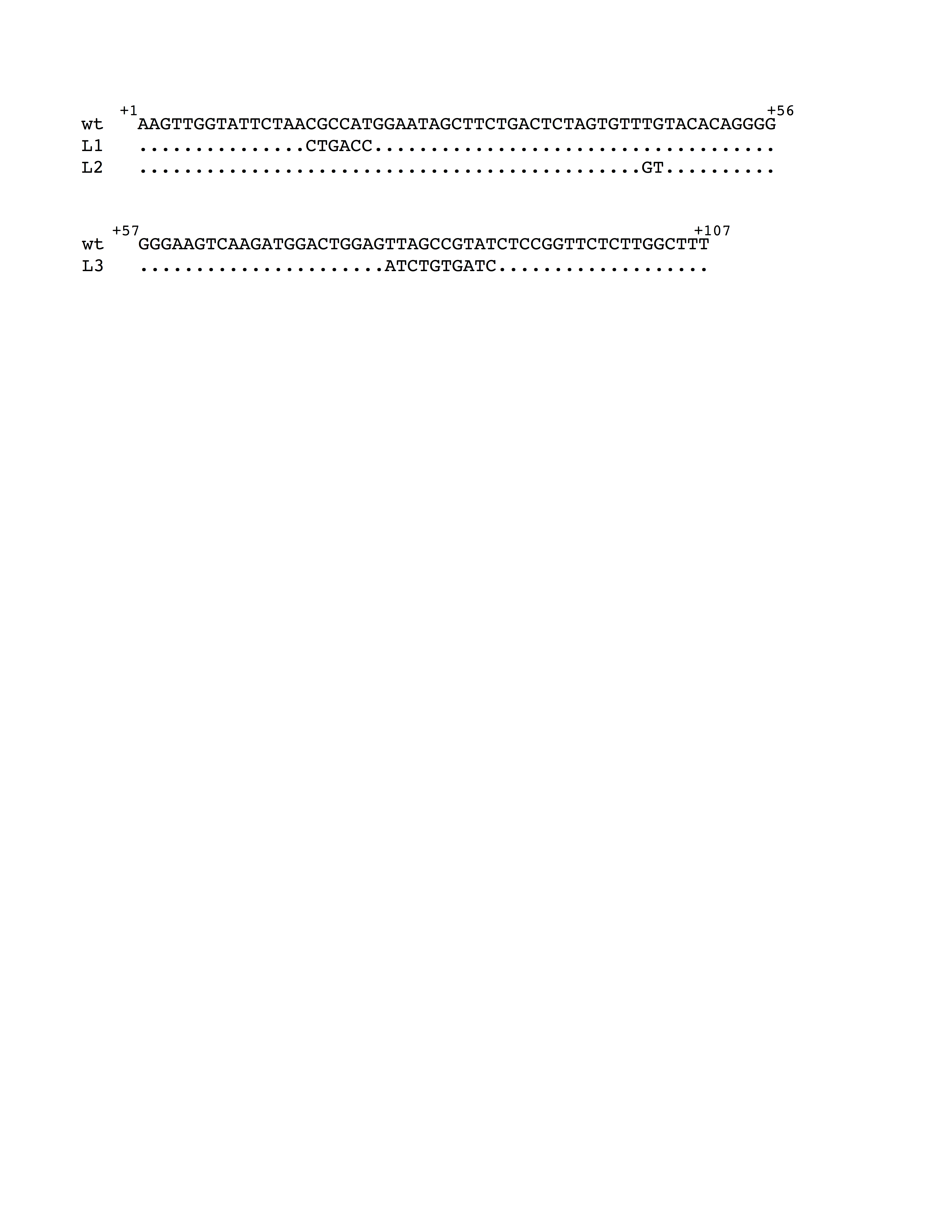

Supplement: S1 Fig — L1, L2 and L3 indicate loop mutants. (TIFF) [file pone.0116593.s001.tiff]

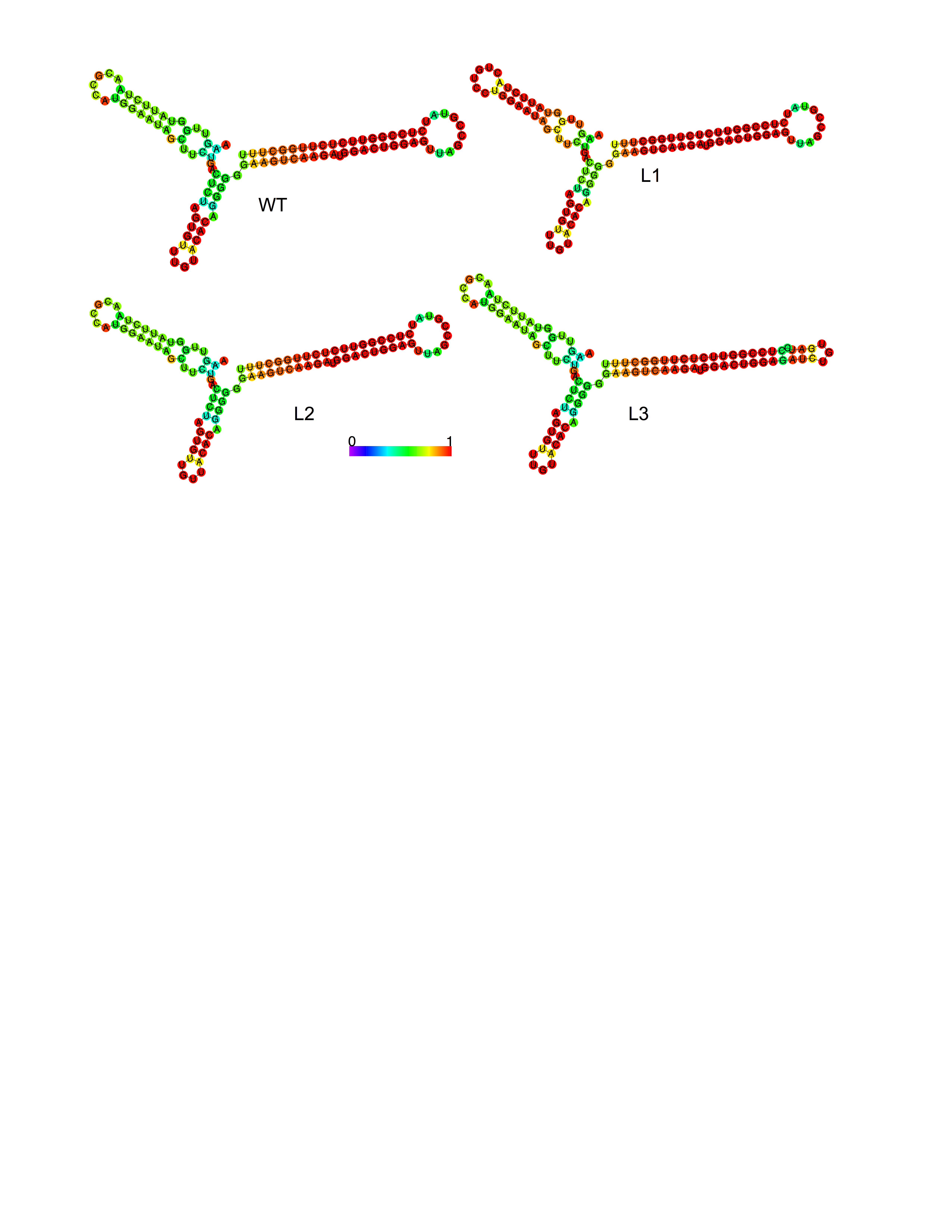

Supplement: S2 Fig — Structure predictions and base pair probabilities (color coded 1–0) were calculated using the RNAfold web server. The predicted structure of wt IhtA is included as a reference. L1, L2 and L3 indicate loop mutants. (TIFF) [file pone.0116593.s002.tiff]

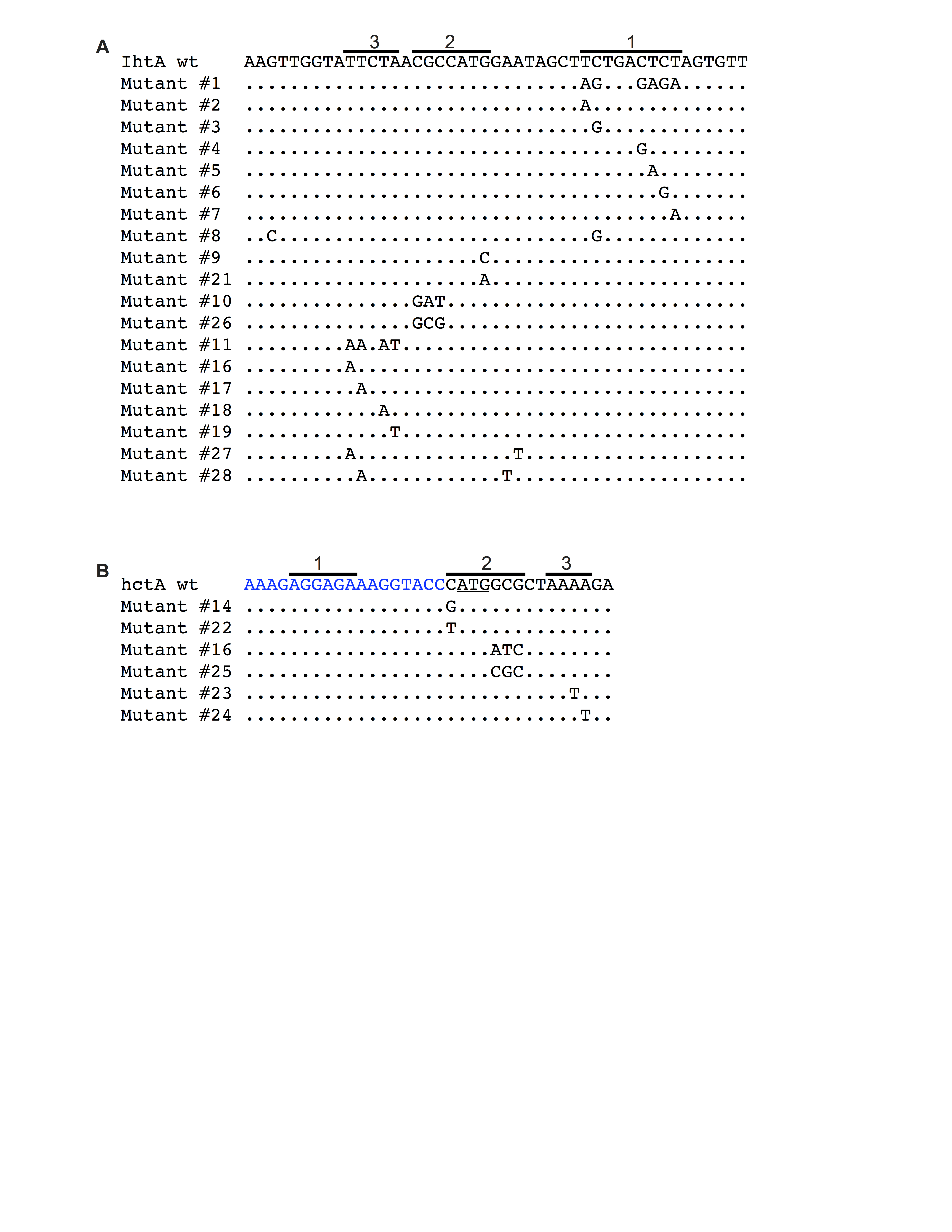

Supplement: S3 Fig — A) IhtA mutants in stem:loop 1. Region 1 (ant-SD), Region 2 (anti-hctA start site) and Region 3 (anti-hctA ORF) are indicated. B) Complementation mutants made in hctA. Regions 1, 2 and 3 are indicated. The blue colored sequence indicates pTet, the Shine-Dalgarno of which is in common with hctA (Region 1). (TIFF) [file pone.0116593.s003.tiff]

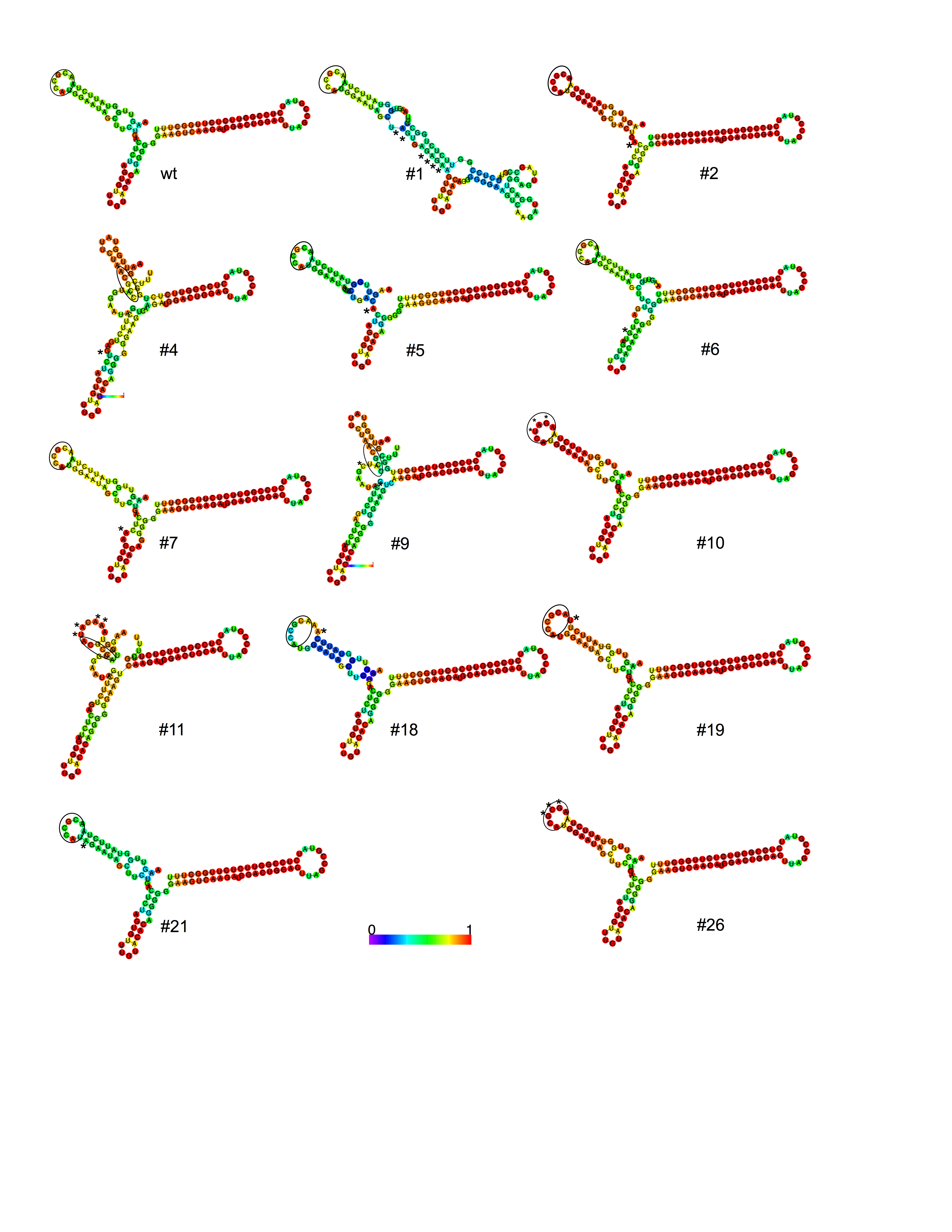

Supplement: S4 Fig — Only structures not included in the manuscript figures are represented here. Structure predictions and base pair probabilities (color coded 1–0) were calculated using the RNAfold web server. All mutations are indicated by an * and the location of the critical G/C clamp is circled. Wild type IhtA and the location of the open loop 1 G/C rich clamp is included as a reference structure. (TIFF) [file pone.0116593.s004.tiff]

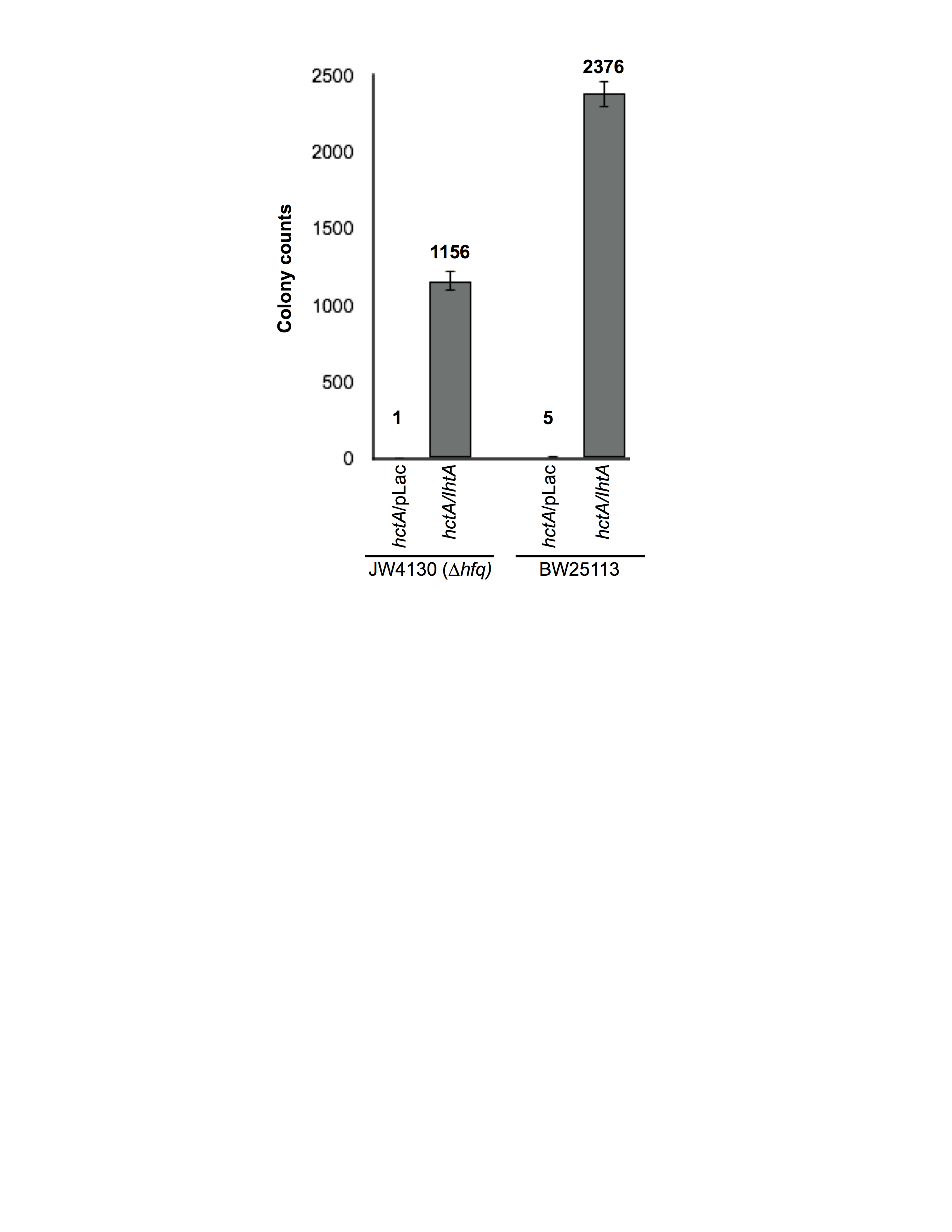

Supplement: S5 Fig — The μhfq strain JW4130–1 generated by Baba et al. in E. coli K12 BW25113 and made available as part of the Keio collection [48], and the parent strain BW25113 were used to verify that IhtA is Hfq independent. Unlike the DH5αPRO E. coli strain generally used for our rescue assays, neither JW4130–1 nor BW25113 express TetR (tetracycline repressor). Therefore transformation of hctA should be lethal in both JW4130–1 and BW25113 as HctA will be constitutively expressed directly upon transformation and few if any colonies should grow. Co-expression of IhtA should rescue this phenotype in both strains if IhtA is Hfq independent but only in BW25113 if Hfq is required. Chemically competent JW4130–1 and BW25113 were co-transformed with either hctApTet+pLac or hctApTet+ihtApLac. Upon transformation and growth at 37°C for an hour, the entire sample was plated on LB agar plates containing the appropriate antibiotics: JW4130–1 transformants were plated on 100 μg/ml cb, 34 μg/ml cm and 25 μg/ml kan, and BW25113 transformants were plated on 100 μg/ml cb and 34 μg/ml cm. The resulting colonies from three separate experiments were counted and graphed. JW4130–1 and BW25113 transformed with hctApTet+pLac resulted in an average of 1 and 5 colonies respectively. When hctApTet+ihtApLac were co-transformed into the Δhfq strain JW4130–1, the average number of colonies increased to 1156, approximately a 1000 fold increase. The average colony count of BW25113 co-transformed with hctApTet+ihtApLac also increased, from 5 to 2376, an approximately 475 fold increase. (TIFF) [file pone.0116593.s005.tiff]

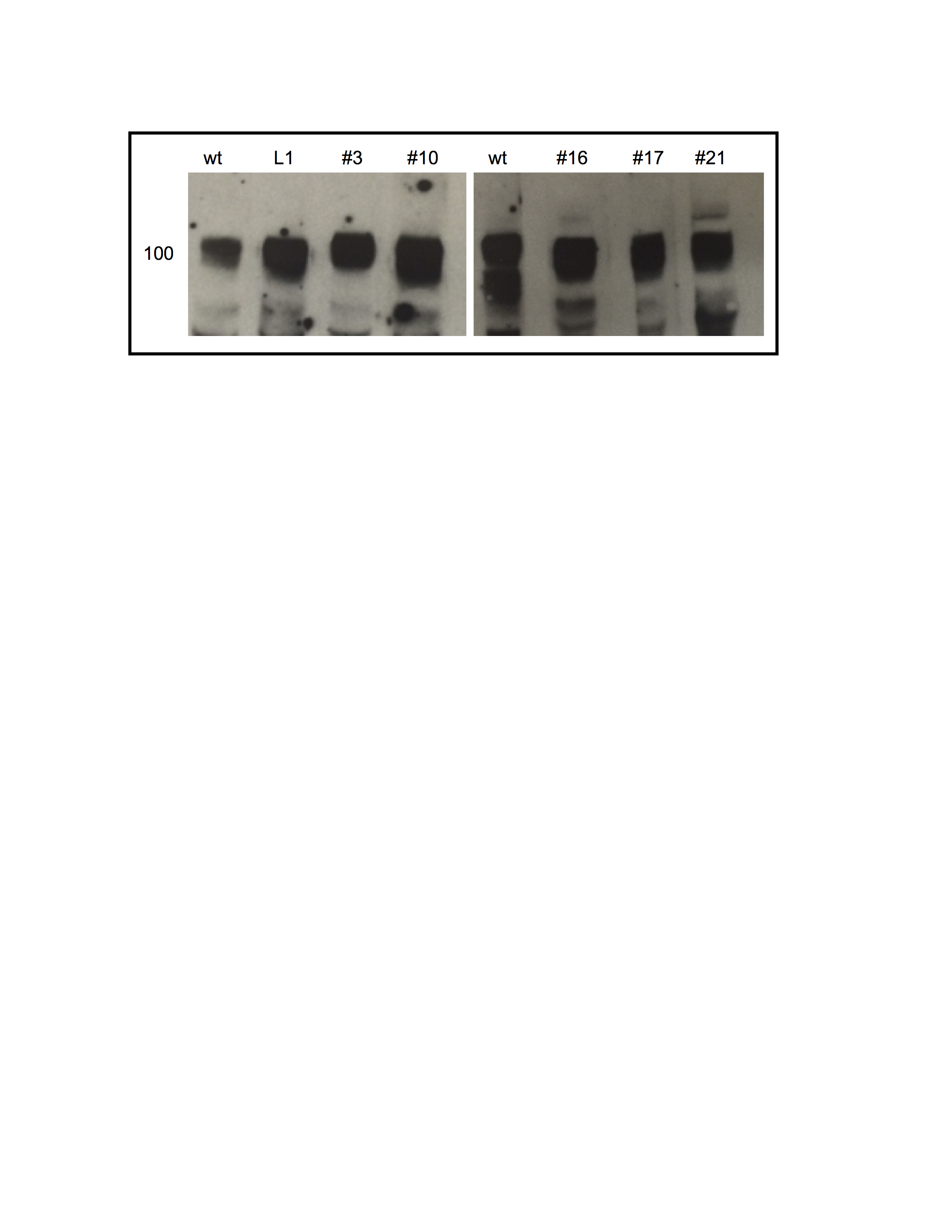

Supplement: S6 Fig — IhtA mutants L1, #3, #10, #16, #17 and #21 were analyzed by Northern blot to ascertain expression levels. These mutants did not rescue wt or mutant HctA induced growth defects. The aforementioned ihtA mutant constructs were grown o/n in LB containing 100 μg/ml cb. E. coli expressing IhtA were pelleted and washed twice in ice cold PBS prior to sRNA isolation using the mirVana miRNA Isolation kit as described by the manufacturer (Ambion, Inc.). Northern analysis was performed on sRNAs separated on a 10% TBE-urea acrylamide gel and transferred to BrightStar-Plus Nylon membrane (Ambion, Inc.). Membranes were hybridized overnight at 42°C in ULTRAhyb with a biotinylated antisense oligo probe designed against the common 3’ end of IhtA (5’ AAAGCCAAGAGAACCGGAGATACGGCTAACTCCAGTCCATCTTGACTTCCCCCCTGTGTAC 3’). The oligo was synthesized by IDT and biotinylated using a BrightStar Psoralen-Biotin Nonisotopic Labeling Kit (Ambion, Inc.). Probed membranes were washed and the IhtA species were detected with the BrightStar BioDetect Nonisotopic detection kit (Ambion, Inc.). (TIFF) [file pone.0116593.s006.tiff]
