# Supplementary material for: Identification of the Base-Pairing Requirements for Repression of hctA Translation by the Small RNA IhtA Leads to the Discovery of a New mRNA Target in Chlamydia trachomatis
Source: PLoS One. 2015 Mar 10;10(3):e0116593. doi: 10.1371/journal.pone.0116593 (PMC4355289; doi:10.1371/journal.pone.0116593)
Supplement: S1 Table — (DOCX) [file pone.0116593.s007.docx]

| **Mutant** | **Primer** |
| --- | --- |
| IhtA#1, F | GTTGGTATTCTAACGCCATGGAATAGCTAGTGAGAGAAGTGTTTGTACACAGGGGGGAAG |
| IhtA#1, R | GACTTCCCCCCTGTGTACAAACACTTCTCTCACTAGCTATTCCATGGCGTTAGAATACCAAC |
| IhtA#2, F | GTACAAACACTAGAGTCAGTAGCTATTCCATGGCGTTAG |
| IhtA#2, R | CTAACGCCATGGAATAGCTACTGACTCTAGTGTTTGTAC |
| IhtA#3, F | CTAACGCCATGGAATAGCTTGTGACTCTAGTGTTTG |
| IhtA#3, R | CCAACACTAGAGTCACAAGCTATTCCATGGCGTTAG |
| IhtA#4, F | TAACGCCATGGAATAGCTTCTGAGTCTAGTGTTTGTAC |
| IhtA#4, R | TAACGCCATGGAATAGCTTCTGAGTCTAGTGTTTGTAC |
| IhtA#5, F | CATGGAATAGCTTCTGACACTAGTGTTTGTACACAGG |
| IhtA#5, R | CCTGTGTACAAACACTAGTGTCAGAAGCTATTCCATG |
| IhtA#6, F | ATGGAATAGCTTCTGACTGTAGTGTTTGTACACAGGG |
| IhtA#6, R | CCCTGTGTACAAACACTACAGTCAGAAGCTATTCCAT |
| IhtA#7, F | GGAATAGCTTCTGACTCAAGTCTTTGTACACAGGG |
| IhtA#7, R | CCCTGTGTACAAACACTTGAGTCAGAAGCTATTCC |
| IhtA#9, F | GTTGGTATTCTAACGCCATCGAATAGCTTCTGACTCTAG |
| IhtA#9, R | GTTGGTATTCTAACGCCATCGAATAGCTTCTGACTCTAG |
| IhtA#10, F | GGAATAATGTTATAAGAGTTGCAAGTTGGTATTCTAAGATCATGGAATAGCTTCTGACTC |
| IhtA#10, R | GAGTCAGAAGCTATTCCATGATCTTAGAATACCAACTTGCAACTCTTATAACATTATTCC |
| IhtA#11, F | AAGCGGAATAATCTTATAAGAGTTGCAAGTTGGTAAACATACGCCATGGAATAGCTTC |
| IhtA#11, R | GAAGCTATTCCATGGCGTATGTTTACCAACTTGCAACTCTTATAACATTATTCCGCTT |
| IhtA#16, F | GTTATAAGAGTTGCAAGTTGGTAATCTAACGCCATGGAATAGC |
| IhtA#16, R | GCTATTCCATGGCGTTAGATTACCAACTTGCAACTCTTATAAC |
| IhtA #17, F | AGAGTTGCAAGTTGGTATACTAACGCCATGGAATAGC |
| IhtA#17, R | AGAGTTGCAAGTTGGTATACTAACGCCATGGAATAGC |
| IhtA#18, F | TATAAGAGTTGCAAGTTGGTATTCAAACGCCATGGAATAGC |
| IhtA#18, R | GCTATTCCATGGCGTTTGAATACCAACTTGCAACTCTTATA |
| IhtA#19, F | GTTATAAGAGTTGCAAGTTGGTATTCTTACGCCATGGAATAG |
| IhtA#19, R | GTTATAAGAGTTGCAAGTTGGTATTCTTACGCCATGGAATAG |
| IhtA#21, F | GTTGGTATTCTAACGCCATAGAATAGCTTCTGACTCTAG |
| IhtA#21, R | CTAGAGTCAGAAGCTATTCTATGGCGTTAGAATACCAAC |
| IhtA#26, F | TAATGTTATAAGAGTTGCAAGTTGGTATTCTAAGCGCATGGAATAGCTTCTGAC |
| IhtA#26, R | GTCAGAAGCTATTCCATGCGCTTAGAATACCAACTTGCAACTCTTATAACATTA |
| IhtA#27, F | GTAATCTAACGCCATGGATTAGCTTCTGACTCTAGTG |
| IhtA#27, R | CACTAGAGTCAGAAGCTAATCCATGGCGTTAGATTAC |
| IhtA#28, F | ACTAGAGTCAGAAGCTATACCATGGCGTTAGTATACC |
| IhtA#28,R | GGTATACTAACGCCATGGTATAGCTTCTGACTCTAGT |
| hctA#14, F | AAGAGGAGAAAGGTACCGATGGCGCTAAAAGATAC |
| hctA#14, R | GTATCTTTTAGCGCCATCGGTACCTTTCTCCTCTT |
| hctA#22, F | AAAGAGGAGAAAGGTACCTATGGCGCTAAAAGATACG |
| hctA#22, R | CGTATCTTTTAGCGCCATAGGTACCTTTCTCCTCTTT |
| hctA#23, F | GAAAGGTACCCATGGCGCTAATAGATACGGCAAA |
| hctA#23, R | TTTGCCGTATCTATTAGCGCCATGGGTACCTTTC |
| hctA#24, F | AAGGTACCCATGGCGCTAAATGATACGGCAAAAAAAATG |
| hctA#24, R | CATTTTTTTTGCCGTATCATTTAGCGCCATGGGTACCTT |
| hctA#25, F | TTAAAGAGGAGAAAGGTACCCATGCGCCTAAAAGATACGGCAAAAAAAATG |
| hctA#25, R | CATTTTTTTTGCCGTATCTTTTAGGCGCATGGGTACCTTTCTCCTCTTTAA |

**Table S1.**Oligos used to generate IhtA and hctA mutants.
