# Supplementary material for: Identification of the Base-Pairing Requirements for Repression of hctA Translation by the Small RNA IhtA Leads to the Discovery of a New mRNA Target in Chlamydia trachomatis
Source: PLoS One. 2015 Mar 10;10(3):e0116593. doi: 10.1371/journal.pone.0116593 (PMC4355289; doi:10.1371/journal.pone.0116593)
Supplement: S2 Table — tThe T7 promoter sequence is underlined. The biotinylated oligos used to immobilize hctA, hctB, CTL0097 and CTL0322 RNA to the BLI biosensor tips are also indicated. (DOCX) [file pone.0116593.s008.docx]

| Primer | Sequence |
| --- | --- |
| T7 Sense IhtA, F | TAATACGACTCACTATAGGGAAGTTGGTATTCTAACGCCATGGAATAGC |
| Sense IhtA, R | AAAGCCAAGAGAACCGGAGATACG |
| T7 Antisense IhtA, F | TAATACGACTCACTATAGGGCCATAAAAAGCCAAGAGAACCGGAGATACG |
| Antisense IhtA, R | AAGTTGGTATTCTAACGCCATGGAATAGC |
| T7 hctA, F | TAATACGACTCACTATAGGGTTAAAACTGAAAAAAATAGTTTAAAACAACAAC |
| hctA, R | TTTTTTTTTTTTTTTTTTTTTTTATTTTTTTGTTGAGCGAGTTTTTTTCGCTG |
| T7 hctB, F | TAATACGACTCACTATAGGGGTAGTAGTTACCTGTCTAATTAGGGGAATG |
| hctB, R | TTTTTTTTTTTTTTTTTTTTTTTTTTCTATCTAGCGACTAATTTCATTAATTGTTGACG |
| T7 CTL0097, F | TAATACGACTCACTATAGGGAGCTAGAGGAGCGGCACGATGGCATCAGAATACG |
| CTL0097, R | TTTTTTTTTTTTTTTTTTTTTTTTTTCTAAAATAATCGGATCAAAGAGCAGCAGAGG |
| T7 CTL0322, F | TAATACGACTCACTATAGGGAGACTTAGTTGACTATTACAATTAATAAAGC |
| CTL0322, R | TTTTTTTTTTTTTTTTTTTTTTTTTTATTCTGTAGGTTGAGAAAGAGCTTCAACG |

Table S2. Primers used to generate IhtA, *hctA, hctB, CTL0097* and *CTL0322* T7 fragments for invitro transcription. The T7 promoter sequence is underlined. The biotinylated oligos used to immobilize hctA, hctB, CTL0097 and CTL0322 RNA to the BLI biosensor tips are also indicated.
