# Supplementary material for: Identification of the Base-Pairing Requirements for Repression of hctA Translation by the Small RNA IhtA Leads to the Discovery of a New mRNA Target in Chlamydia trachomatis
Source: PLoS One. 2015 Mar 10;10(3):e0116593. doi: 10.1371/journal.pone.0116593 (PMC4355289; doi:10.1371/journal.pone.0116593)
Supplement: S3 Table — (DOCX) [file pone.0116593.s009.docx]

| Primer | Sequence |
| --- | --- |
| CheZ Transfer, F (∆cheZ) | AGCCATTTACCGCCGCGACGCTGGAGG |
| CheZ Transfer, R (∆cheZ) | ACATCAAGCATAGTGTGCGTTTGTCG |
| CheZ-pTet plasmid, F | CAACCATCAATCAAACCTGCTGACGAGC |
| CheZ-pTet plasmid, R | GGGTACCTTTCTCCTCTTTAATGAATTC |
| pTet/CTL0322 F, infusion | GAGGAGAAAGGTACCCaagcaaaaactaccatctgtagaac |
| CTL0322/cheZ R, infusion | GTTTGATTGATGGTTGttcttggatagtaatctgtgccg |
| pTet/CTL0097 F, infusion | GAGGAGAAAGGTACCCAAACGGTAAGATAACCTTATAGTG |
| CTL0097/cheZ R, infusion | GTTTGATTGATGGTTGTTACCGAAGTGCTCGTATTCTG |
| pTet/hctB F, infusion | GAGGAGAAAGGTACCCGTAGTAGTTACCTGTCTAATTAGG |
| hctB/cheZ R, infusion | GTTTGATTGATGGTTGGCGTTTCTTTTGTACTCCCAAC |
| pTet/hctA F, infusion | gaggagaAAGGTACCCattaaaactgaaaaaaatagtttaaaac |
| hctA/cheZ R, infusion | GTTTGATTGATGGTTGCGCCATGAAAAAATATCCTCTAG |
| pTet/cheZ F, infusion | TTAAAGAGGAGAAAGGTACCcATGATGCAACCATCAATCAAACCTGC |
| cheZ/pTet R, infusion | TCGGGCCCTGAGGCCTGCAgTCAAAATCCAAGACTATCCAACAAATCG |

Table S3. Primers used to generate the cheZ deletion in strain MG1655 (CheZ Transfer, F and CheZ Transfer, R) and the clones used in the CheZ motility assay.
